# Synthesis of Novel 1-(4-Substituted pyridine-3-sulfonyl)-3-phenylureas with Potential Anticancer Activity

**DOI:** 10.3390/molecules200712029

**Published:** 2015-07-01

**Authors:** Krzysztof Szafrański, Jarosław Sławiński

**Affiliations:** Department of Organic Chemistry, Medical University of Gdańsk, Al. Gen. J. Hallera 107, 80-416 Gdańsk, Poland; E-Mail: k.szafranski@gumed.edu.pl

**Keywords:** sulfonamides, pyridine-3-sulfonamides, sulfonylureas, diarylsulfonylureas, anticancer, antitumor activity

## Abstract

A series of novel 4-substituted-*N*-(phenylcarbamoyl)-3-pyridinesulfonamides **11**–**27** have been synthesized by the reaction of 4-substituted pyridine-3-sulfonamides **2**–**10** with the appropriate aryl isocyanates in presence of potassium carbonate. The *in vitro* anticancer activity of compounds **11**, **12**, **14**–**21** and **24**–**26** was evaluated at the U.S. National Cancer Institute and in light of the results, some structure-activity relationships were discussed. The most prominent compound, *N*-[(4-chlorophenyl)carbamoyl]-4-[4-(3,4-dichlorophenyl)piperazin-1-yl]pyridine-3-sulfonamide (**21**) has exhibited a good activity profile and selectivity toward the subpanels of leukemia, colon cancer and melanoma, with average GI_50_ values ranging from 13.6 to 14.9 µM.

## 1. Introduction

Sulfonylureas have been used in pharmacotherapy since the 1950s when the first antidiabetic compound *carbutamide* was released to the market. Since then, several sulfonylureas have been commonly used as antihypoglycemic (e.g., *tolbutamide*, *glipizide*, *glimepiride*), diuretics (*torasemide*) or herbicides (*rimsulfuron*). Large numbers of sulfonylurea derivatives have also been tested in preclinical studies among others towards histamine H_3_ receptor antagonism [1,2], thromboxane A_2_ receptor antagonism [3,4], antibacterial [5], antimalarial [6] or antifungal activity [7], and in particular, an important part of this research concerns antitumor activity [8,9,10,11,12]. 

**Figure 1:**
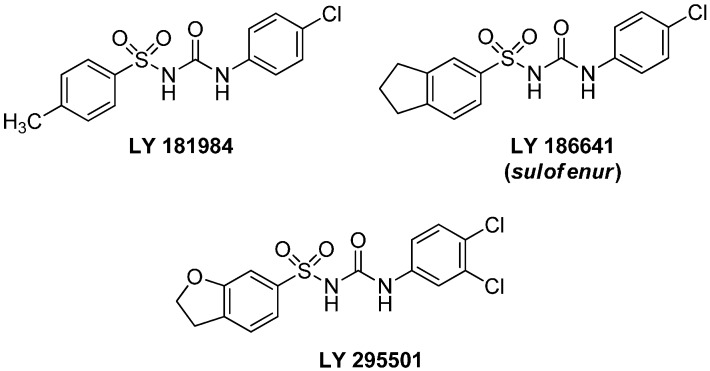
Anticancer diarylsulfonylureas (DSUs).

Studies on the antineoplastic activity led to discovery of *N*^1^,*N*^3^-diarylsulfonylureas (DSUs): LY-181984, LY-186641 (*sulofenur*) and LY-295501 (Figure 1) which were recognized as a group of compounds with activity against a broad spectrum of syngeneic rodent solid tumors and human tumor xenografts [13].

Due to the high *in vivo* activity in mouse models, *sulofenur* and LY-295501 have been evaluated in phase I or II clinical trials on patients with a non-small cell lung carcinoma (NSCLC), renal carcinoma. melanoma, and ovarian cancer [14]. Despite the disappointing results of the tests, mainly due to high hemotoxicity, novel sulfonylurea derivatives with anticancer activity are constantly being developed [15].

**Figure 2:**
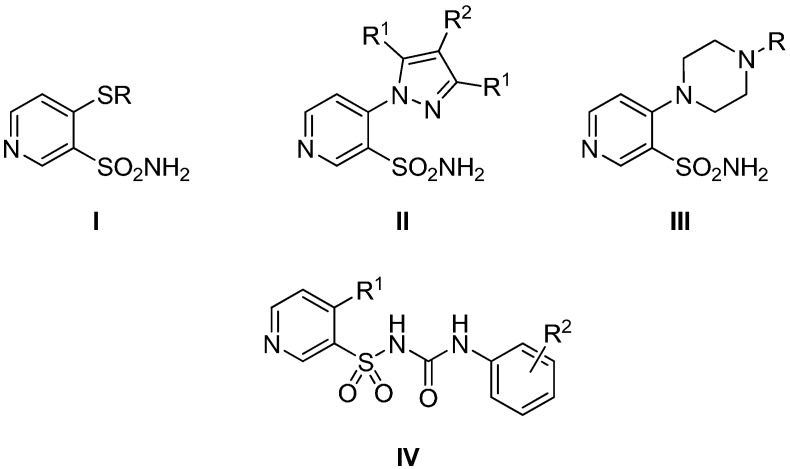
General structure of 4-substituted pyridine-3-sulfonamide derivatives.

Recently, we have reported on the synthesis of a series of 4-substituted pyridine-3-sulfonamides bearing primary sulfonamide moieties [16,17] as an efficient and selective carbonic anhydrase inhibitors (types **I**–**III**, Figure 2), whereas 4-piperazin-1-yl-pyridine-3-sulfonamides of type **III** (Figure 2) exhibited also moderate anticancer activity [17]. It is also well known that selective hCA inhibitors have a proven ability to limit the growth and restrict metastasis in xenograft models of breast cancer [18,19], and therefore they are considered as novel type of anticancer drugs with novel mechanism of action [20,21,22,23,24].

These findings prompted us to explore further series of DSUs possessing 1-(4-substituted pyridine-3-sulfonyl)-3-phenylureas core of type **IV** (Figure 2), and to investigate their anticancer properties.

## 2. Results and Discussion

### 2.1. Chemistry

**Scheme 1:**
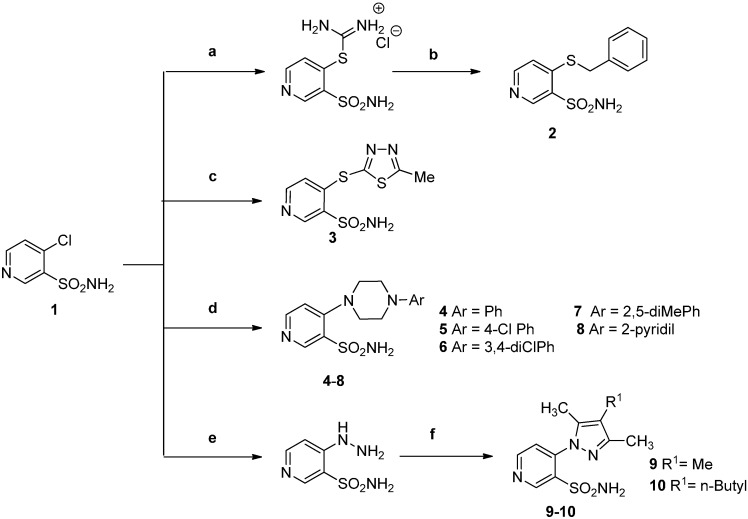
Facile synthesis of the starting 4-substituted 3-pyridinesulfonamide derivatives **2**–**10**.

The starting 4-substituted pyridine-3-sulfonamides **2**, **4**–**10** were prepared according to the known methods [16,17], while the novel compound 4-[(5-methyl-1,3,4-thiadiazol-2-yl)thio]-3-pyridine-sulfonamide (**3**) was synthesized by the reaction of aromatic nucleophilic substitution of chlorine atom of **1** with 5-methyl-1,3,4-thiadiazole-2-thiol, under forcing conditions of elevated pressure and temperature (Scheme 1).

*Reagents and conditions*: (**a**) thiourea (1.02 eq.), MeCN, reflux, 3 h, [16]; (**b**) NaOH (2.14 eq.) water/THF 5 °C, 0.5 h, r.t. 5 h [16]; (**c**) 5-methyl-1,3,4-thiadiazole-2-thiol (1.2 eq.), MeCN r.t. 2 h, 100 °C 24 h, elevated pressure; (**d**) 4-Ar-piperazine (2 eq.), MeOH, r.t. 28 h, or 4-Ar-piperazine hydrochloride (1.2 eq.), Et_3_N (2.25 eq.), MeOH r.t. 72 h [17]; (**e**) H_2_NNH_2_ × H_2_O (6 eq.), MeOH r.t. 16 h, reflux, 4 h [16]; (**f**) 3-R^1^-2,4-pentanedione (1.1 eq.), AcOH, reflux, 2 h [17].

**Scheme 2:**
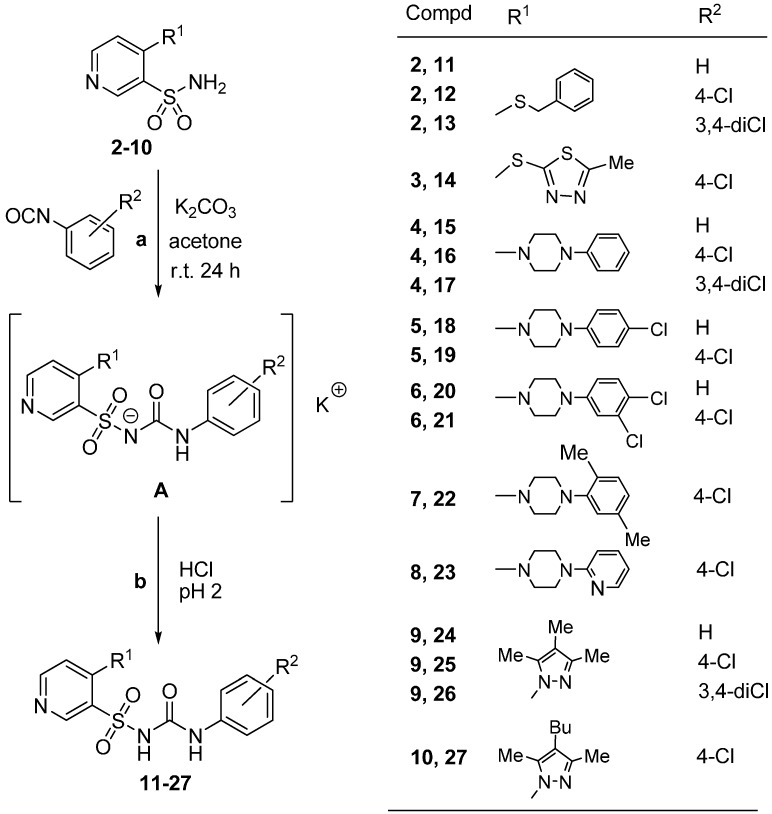
Synthesis of 4-substituted-*N*-(R^2^-phenylcarbamoyl)-3-pyridinesulfonamides **11**–**27**.

The desired, 4-substituted *N*-(phenylcarbamoyl)pyridine-3-sulfonamides **11**–**27** were obtained by treatment for 24 h of primary sulfonamides **2**–**10** with the appropriate aryl isocyanates (*i.e.*, phenyl isocyanate, 4-chlorophenyl isocyanate or 3,4-dichlorophenyl isocyanate) in dry acetone at room temperature in the presence of anhydrous potassium carbonate (Scheme 2). Then, the initially formed intermediate potassium salts of type **A** were acidified with dilute hydrochloric acid to pH 2, to afford expected diarylsulfonylurea products in moderate to good yields (47%–80%).

*Reagents and conditions*: (**a**) arylisocyanate (1.2 eq.), anhydrous K_2_CO_3_ (1.0 eq.), dry acetone, r.t. 24 h; (**b**) H_2_O 5 mL, 4% HCl pH 2 r.t. 16 h.

The final compounds were characterized by IR and NMR spectroscopy as shown in the Experimental Section. Elemental analyses (C,H,N) were in accordance with the proposed structures. In detail, IR absorption bands corresponding to the stretching vibration of the carbonyl group in -NH(C=O)NH- appeared for compounds **11**–**14** and **24**–**27** in the range of 1716–1732 cm^−1^, and was shifted to 1630–1648 cm^−1^ for compounds **15**–**23** possessing a piperazine ring attached at the 4 position. Inspection of the ^1^H-NMR spectra revealed the characteristic resonance signals of pyridine ring protons appeared as two doublets of H-5 and H-6 at 7.18–7.64 and 8.27–8.96 ppm, respectively, as well as a singlet signal of H-2 at 8.71–9.26 ppm. In turn, the singlet signals attributable to the NH protons of the urea moieties were observed at 8.9–9.4 ppm, whereas a broad singlet of the SO_2_NH proton was found downfield in the wide region of 9.7–13.4 ppm.

### 2.2. Anticancer Activity

Compounds **11**, **12**, **14**–**21** and **24**–**26** were tested *in vitro* at the U.S. National Cancer Institute (Bethesda, MD, USA) at single high concentration of 10 µM against the NCI panel of 60 cell lines derived from nine different human cancer types: leukemia, non-small-cell lung cancer (NSCLC), colon, central nervous system (CNS), melanoma, ovarian, renal, prostate and breast cancer. Inhibition growth percent (IGP) data compared to no-drug control, obtained for each of the cell lines, are shown in Table 1.

**Table 1:** Inhibition growth percent (IGP [%]) for tested compounds (**11**, **12**, **14**–**21**, **24**–**26**) against selected NCI-60 cancer cell lines at single concentration 10^−5^ M.^a^

Panel	Cell Line	IGP [%] of Compound												
		11	12	14	15	16	17	18	19	20	21	24	25	26
*Leukemia*	CCRF-CEM	1	4	27	30	7	–	2	11	*	85	27	19	–
	K-562	6	67	–	45	–	7	12	70	20	95	42	72	*
	MOLT-4	7	21	–	27	–	6	5	18	9	85	9	20	8
	RPMI-8226	*	*	–	70	–	*	1	7	*	93	72	10	*
	SR	–	15	–	16	–	12	–	–	–	95	1	29	19
*NSCLC*	HOP-92	*	*	–	53	–	*	*	2	*	131	28	*	–
	NCI-H522	7	25	*	43	*	8	9	19	5	110	28	12	8
*Colon cancer*	HCC-2998	11	1	*	1	*	*	11	9	2	73	*	2	*
	HCT-15	*	39	37	*	31	9	*	28	*	41	*	53	4
	KM12	*	43	*	25	4	7	–	24	14	89	27	49	*
	SW-620	*	24	30	10	21	*	*	30	*	82	12	40	*
*CNS cancer*	SF-268	*	18	3	1	6	*	*	19	6	71	*	26	*
	SF-295	11	13	6	43	13	10	*	10	–	147	48	*	1
*Melanoma*	LOX IMVI	7	-	59	10	56	–	3	47	4	96	10	59	–
	MALME-3M	9	1	*	58	*	3	6	6	5	101	44	2	*
	M14	*	17	16	27	17	*	*	9	0	77	25	29	*
	MDA-MB-435	*	9	16	11	12	*	*	*	*	93	15	23	*
	UACC-62	*	57	51	33	38	*	*	32	*	73	31	42	10
*Ovarian cancer*	OVCAR-3	*	*	*	42	*	*	*	*	*	75	50	*	*
*Renal cancer*	A498	15	19	33	4	42	5	17	13	12	80	7	6	0
	ACHN	*	31	22	25	21	*	*	24	*	93	26	25	*
	CAKI-1	2	33	29	*	28	11	*	23	*	65	5	17	2
	UO-31	12	18	17	27	19	9	5	21	10	110	20	29	16
*Prostate cancer*	PC-3	11	6	14	54	17	*	6	21	16	101	51	12	*
	DU-145	*	16	5	*	7	*	*	18	*	67	1	32	*
*Breast cancer*	MDA-MB-468	*	7	–	59	–	3	*	*	*	109	57	*	*
	T-47D	2	20	21	33	17	6	10	27	9	92	26	18	7

^a^ Data obtained from NCI-60 DTP human tumor cell line screening [25,26,27,28,29], * IGP ≤ 0%; – not tested.

The most important conclusion based on this preliminary anticancer assay is the finding that substitution patterns of the urea phenyl ring (R^2^), has a crucial impact on the cytotoxic activity potency:

(a) All compounds with 4-chlorophenylcarbamoyl (R^2^ = 4-Cl) moieties (**12**, **14**, **16**, **19**, **21** and **25**) are characterized by moderate to high activity with IGP range from 17 to 96% against the common cancer cell lines: leukemia (K-562, MOLT-4), colon cancer (HCT-15 and SW-620,) melanoma (LOX IMVI and UACC-62), and renal cancer (ACHN, CAKI-1) (Table 1), as well exhibit high overall activity with the average IGP for the whole panel in the range from 7 to 90%.
(b) Apparently the introduction of a second chlorine atom in position 3 of the 4-chloro-phenylcarbamoyl moiety (R^2^ = 3,4-diCl) in compounds **17** and **26** definitely causes a loss of activity. In this case the highest IGP values of 12 and 19% are observed only for the leukemia SR cell line.
(c) From among of the compounds with unsubstituted phenylcarbamoyl moieties (R^2^ = H; *i.e.*, **11**, **15**, **18**, **20** and **24**) only compounds **15** and **24** exhibit high antiproliferative activity against certain cell lines: leukemia RPMI-8226 (IGP = 70 and 72%), NSCLC HOP-92 (IGP = 53 and 28%), CNS cancer (IGP = SF-295 43 and 48%), melanoma MALME-3M (IGP = 58 and 44%), ovarian cancer OVCAR-3 (IGP = 42 and 50%), prostate cancer PC-3 (IGP = 54 and 51%) or breast cancer MDA-MB-468 (IGP = 59 and 57%), respectively (Table 1). It should be noted that the mentioned cell lines, which are highly susceptible for compounds **15** and **24**, do not exhibit significant sensitivity for their 4-chlorophenylurea (R^2^ = 4-Cl) analogs **16** and **25**.

It was stated that among a series of 4-chlorophenylureas (**12**, **14**, **16**, **19**, **21** and **25**) replacement of the benzylthio moiety (R^1^) at the 4 position of the pyridine ring (compound **12**) by a 5-methyl-1,3,4-thiadiazole-2-thiol group in **14** led to a decrease of the susceptibility of NCI-H522 (from 25 to 0%), KM-12 (from 43 to 0%), however the cell lines CCRF-CEM, HCT-116 remain more sensitive to compound **12** (increase from 0 to 25% and from 0 to 43%, respectively). On the other hand removal of the sulfur atom and introduction of a pyrazole moiety in **25** leads to higher activity towards the majority of cell lines, especially for HCT-15, KM 12, SF-268, UO-31 and DU-145 (Table 1).

Additionally, introduction of a chlorine atom in **19** to the phenylpiperazinyl substituent of **16** results in a slight increase of antiproliferative activity towards the NSCLC cell line NCI-H522 (from 0 to 19%), colon cancer HCC-2998 (from 0 to 9%), KM12 (from 4 to 24%), SW-620 (from 21 to 30%), CNS cancer SF-268 (from 6 to 19%), prostate cancer PC-3 (from 17 to 21%), DU-145 (from 7 to 18%) breast cancer T-47D (from 17 to 27%), however it also resulted in loss of activity against renal cancer A498 (from 42 to 13%) melanoma M14 (from 17 to 9%) and MDA-MB-435 (from 12 to 0%) (Table 1). Nevertheless introducing of second chlorine atom at position 3 of the 4-chlorophenylpiperazin-1-yl in **21** causes a huge increase of activity since all mentioned lines demonstrate IGPs over 66%. The average IGP for all cell lines reached 90% and furthermore a cytotoxic effect (IGP > 100%) was observed for sixteen cell lines representing all nine cancer types.

Thus, the most active compound *N*-[(4-chlorophenyl)carbamoyl]-4-[4-(3,4-dichlorophenyl)-piperazin-1-yl]pyridine-3-sulfonamide (**21**) was further tested at the NCI five-dose assay (in the range of 0.01–100 µM) (Table 2). A relatively highest sensitivity to this compound was found for the subpanels of leukemia, colon cancer and melanoma, which the average GI_50_ values range from 13.6 to 14.9 µM (*sulofenur* (NSC-642684) 25.5–37.5 µM [30]).

Thus, the lowest GI_50_ was found for the cell lines of melanoma UACC-62 (1.5 µM) and M14 (8.9 µM), leukemia K-562 (3 µM) and CCRF-CEM (8.2 µM), and colon cancer HCT-15 (6.4 µM) as well as for the renal cancer CAKI-1 (6.28 µM) and UO-31 (9.8 µM) cell lines. Furthermore the GI_50_ values for the most sensitive cell lines (GI_50_ < 20 µM) for compound **21** are of the same order of magnitude and generally lower than for clinically tested *sulofenur* (Table 2).

**Table 2:** Anticancer *in vitro* data for compound **21** for the most sensitive cell lines (GI_50_ < 20 µM) ^a^, and comparative data for *sulofenur* (NSC-642684) ^b^ [30].

Panel	Cell line	21			Sulofenur ^b^		
		GI_50_^c^	TGI ^d^	LC_50_^e^	GI_50_^c^	TGI ^d^	LC_50_^e^
*Leukemia*	CCRF-CEM	8.2	43.6	>100	29.8	>100	>100
	K-562	3.0	>100	>100	10.2	77.1	>100
	MOLT-4	19.9	68.1	>100	26.4	98.2	>100
	RPMI-8226	14.7	54.2	>100	24.4	85.7	>100
	SR	11.8	51.6	>100	57.0	>100	>100
*NSCLC*	A-549/ATCC	14.7	62.8	>100	32.7	97.7	>100
	HOP-62	18.8	43.5	>100	32.2	>100	>100
	NCI-H226	13.4	57.9	>100	37.9	77.8	>100
	NCI-H23	19.9	51.9	>100	28.1	56.0	90.8
	NCI-H322M	18.3	>100	>100	34.2	95.5	>100
	NCI-H460	17.2	69.4	>100	38.3	100	>100
	NCI-H522	14.1	49.4	>100	27.9	94.2	>100
*Colon cancer*	COLO 205	16.8	33.0	65.0	29.8	60.8	90.2
	HCC-2998	15.5	31.6	64.3	31.6	77.6	98.4
	HCT-116	15.1	34.4	78.2	26.2	67.3	85.1
	HCT-15	6.4	68.3	>100	35.6	90.2	>100
	HT29	15.9	45.7	>100	61.1	>100	>100
	KM12	13.0	>100	>100	36.1	98.9	>100
	SW-620	14.4	>100	>100	35.3	>100	>100
*CNS cancer*	SF-295	12.2	37.9	>100	46.7	>100	>100
	SF-539	13.1	29.0	64.4	28.3	69.2	>100
	SNB-19	19.1	57.6	>100	40.9	>100	>100
	U251	13.0	36.5	>100	30.7	77.4	>100
*Melanoma*	M14	8.9	53.4	>100	37.8	97.5	>100
	MDA-MB-435	18.9	>100	>100	17.6	48.3	>100
	SK-MEL-5	10.9	36.7	>100	26.8	74.0	98.2
	UACC-62	1.5	15.5	>100	23.9	84.7	>100
*Ovarian cancer*	IGROV1	16.3	68.1	>100	12.3	94.4	>100
	OVCAR-3	16.6	36.7	81.1	24.3	73.8	>100
	OVCAR-8	18.6	>100	>100	40.8	>100	>100
	NCI/ADR-RES	18.4	>100	>100	–	–	–
*Renal cancer*	786-0	16.1	43.7	>100	35.3	>100	>100
	A498	15.2	63.1	>100	45.0	>100	>100
	ACHN	11.6	>100	>100	29.4	>100	>100
	CAKI-1	6.3	>100	>100	42.8	>100	>100
	SN12C	18.4	>100	>100	39.2	>100	>100
	UO-31	9.8	27.3	75.0	25.1	76.4	>100
*Prostate cancer*	PC-3	14.4	71.1	>100	19.0	47.2	>100
	DU-145	17.3	55.7	>100	24.0	92.0	>100
*Breast cancer*	MCF7	19.7	97.4	>100	24.1	81.8	>100
	MDA-MB-231/ATCC	14.6	63.3	>100	–	–	–
	BT-549	16.1	44.7	>100	32.6	>100	>100
	T-47D	13.9	87.6	>100	16.4	74.6	>100
	MDA-MB-468	18.9	>100	>100	–	–	–

^a^ Data obtained from NCI-60 DTP human tumor cell line screening [25,26,27,28,29]; ^b^ Sulofenur (NSC-642684): NCI cancer screen; September 2014 [30]; ^c^ GI_50_: molar concentration [µM] that inhibits 50% net cell growth; ^d^ TGI: molar concentration [µM] giving total growth inhibition; ^e^ LC_50_: molar concentration [µM] causing 50% net cell death; – not tested.

## 3. Experimental Section

### 3.1. General Procedures

The following instruments and parameters were used: melting points Boetius HMK apparatus; IR spectra: KBr pellets, 400–4000 cm^−1^ Thermo Mattson Satellite FTIR spectrophotometer; ^1^H- and ^13^C-NMR: Varian Gemini 200 apparatus at 200 MHz (^1^H-NMR) and 50 MHz (^13^C-NMR), Varian Unity 500 Plus apparatus at 500 MHz (^1^H-NMR) and 125 MHz (^13^C-NMR); chemical shifts are expressed in parts per million (ppm) relative to TMS as an internal standard. Elemental analyses were performed on PerkinElmer 2400 Series II CHN Elemental Analyzer, and they were in agreement with the theoretical values within ±0.4% range. The starting 4-chloro-3-pyridinesulfonamide (**1**) was commercially available (Alfa Aesar, Karlsruhe, Germany), while 4-substituted 3-pyridinesulfonamide **2**, **4**–**10** were obtained according to methods described previously: 4-benzylthio-3-pyridinesulfonamie (**2**) [16], 4-(4-substituted-piperazin-1-yl)-3-pyridinesulfonamides **4**–**8** [17], and 4-(1*H*-pyrazol-1-yl)-3-pyridine-sulfonamides **9**, **10** [17].

### 3.2. Synthesis

#### 3.2.1. Procedure for the Preparation of 4-[(5-Methyl-1,3,4-thiadiazol-2-yl)thio]-3-pyridinesulfonamide (**3**)

In a closed glass pressure tube, a mixture of 4-chloro-3-pyridinesulfonamide (1.92 g, 10 mmol) and 5-methyl-1,3,4-thiadiazole-2-thiol (1.57 g, 12 mmol) in dry acetonitrile (16.5 mL) was stirred at room temperature for 2 h and then at 100 °C for 24 h. The precipitate of the hydrochloride was collected by filtration, washed with acetonitrile (3 × 2 mL) and dried, then suspended in water (8 mL) and slowly adjusted to pH 8 with 1% solution of NaOH. After stirring at room temperature for 3 h, the precipitate was filtered off, washed with water (2 × 1 mL), and dried. Yield 2.49 g (86%); mp 132–135 °C; IR (KBr) ν_max_ 3546, 3344 (NH), 1572 (C=C), 1330, 1163 (SO_2_) cm^−^^1^; ^1^H-NMR (DMSO-*d*_6_, 200 MHz) δ: 2.83 (s, 3H, CH_3_); 7.09 (d, 1H, H-5 pyrid.); 8.00 (s, 2H, NH_2_); 8.56 (d, 1H, H-6 pyrid.); 8.95 (s, 1H, H-2 pyrid.) ppm; anal. C 33.32, H 2.80, N 19.43% calcd. for C_8_H_8_N_4_O_2_S_3_ C 33.02, H 2.84, N 19.33%.

#### 3.2.2. General Procedure for the Preparation of 4-Substituted-*N*-(R^2^-phenylcarbamoyl)-3-pyridine-sulfonamides

A mixture of 4-substituted-3-pyridinesulfonamide **2**–**10** (1 mmol), the appropriate phenyl isocyanate (1.2 mmol) and anhydrous potassium carbonate (0.14 g, 1 mmol) in dry acetone (15 mL) was stirred at room temperature for 24 h. The precipitate containing potassium salt of type **A** (Scheme 2) and inorganic salts was collected by filtration and washed with acetone (2 × 5 mL). The solid thus obtained was suspended in water (5 mL), slowly acidified with 4% hydrochloric acid to pH 2, and stirred overnight. The precipitate was filtered off washed with water (2 × 2 mL), and dried. In this manner the following compounds were obtained:

*4-(Benzylthio)-N-(phenylcarbamoyl)-3-pyridinesulfonamide* (**11**). Starting from 4-benzylthio-3-pyridinesulfonamide (**2**, 0.28 g), and phenyl isocyanate (0.14 g), the title compound **11** was obtained (0.32 g, 80%): mp 198–201 °C; IR (KBr) ν_max_ 3369 (NH), 3081 (C_Ar_-H) 2928, 2853 (C-H), 1727 (C=O), 1600, 1579, 1547 (C=C, C=N), 1350, 1160 (SO_2_) cm^−1^; ^1^H-NMR (DMSO-*d*_6_, 200 MHz) δ 4.51 (s, 2H, CH_2_), 7.03 (t, 1H, H-4ʹ Ph), 7.18–7.34 (m, 7H, H_Ar_), 7.47 (m, 2H, H_Ar_), 7.64 (d, *J* = 5.5 Hz, 1H, H-5 pyrid.), 8.58 (d, *J* = 5.5 Hz, 1H, H-6 pyrid.), 8.71 (s, 1H, H-2 pyrid.), 8.91 (s, 1H, NH), 11.0 (br.s, 1H, NH) ppm; ^13^C-NMR (DMSO-*d*_6_, 125 MHz) δ 35.52, 119.47, 121.45, 124.06, 128.31, 129.29, 129.61, 129.87, 131.89, 135.62, 138.43, 149.58, 149.67, 150.95, 152.89 ppm; anal. C 57.12, H 4.29, N 10.52% calcd for C_19_H_17_N_3_O_3_S_2_ C 56.77, H 4.26, N 10.48%.

*4-(Benzylthio)-N-[(4-chlorophenyl)carbamoyl]-3-pyridinesulfonamide* (**12**). Starting from 4-benzyl-thio-3-pyridinesulfonamide (**2**, 0.28 g), and 4-chlorophenyl isocyanate (0.18 g), the title compound **12** was obtained (0.25 g, 58%): mp 204–207 °C; IR (KBr) ν_max_ 3391 (NH), 3107 (C_Ar_-H) 2851 (C-H), 1725 (C=O), 1599, 1581, 1539, 1494 (C=C, C=N), 1347, 1153 (SO_2_) cm^−1^; ^1^H-NMR (DMSO-*d*_6_, 200 MHz) δ 4.50 (s, 2H, CH_2_), 7.24 (m, 3H, H_Ar_), 7.33 (s, 4H, H_Ar_), 7.46 (m, 2H, H_Ar_), 7.64 (d, *J* = 5.0 Hz, 1H, H-5 pyrid.), 8.58 (d, *J* = 5.0 Hz, 1H, H-6 pyrid.), 8.85 (s, 1H, H-2 pyrid.), 8.90 (s, 1H, NH), ppm; ^13^C-NMR (DMSO-*d*_6_, 125 MHz) δ 35.52, 121.13, 121.47, 127.57, 128.32, 129.28, 129.43, 129.89, 131.98, 135.61, 137.53, 149.85, 150.74, 152.68 ppm; anal. C 52.59, H 3.72, N 9.68% calcd for C_19_H_16_ClN_3_O_3_S_2_ C 52.21, H 3.60, N 9.78%.

*4-(Benzylthio)-N-[(3,4-dichlorophenyl)carbamoyl]-3-pyridinesulfonamide* (**13**). Starting from 4-benzylthio-3-pyridinesulfonamide (**2**, 0.28 g), and 3,4-dichlorophenylisocyanate (0.23 g), the title compound **13** was obtained (0.27 g, 57%): mp 205–208 °C; IR (KBr) ν_max_ 3377 (NH), 3093 (C_Ar_-H), 2924, 2852 (C-H), 1732 (C=O), 1599, 1578, 1532, 1477 (C=C, C=N), 1352, 1155 (SO2) cm^−1^; ^1^H-NMR (DMSO-*d*_6_, 200 MHz) δ 4.50 (s, 2H, CH_2_), 7.24–7.30 (m, 4H, H_Ar_), 7.44–7.52 (m. 3H, H_Ar_), 7.66–7.68 (m, 2H, H_Ar_, H-5 pyrid_._), 8.58 (d, 1H, H-6 pyrid.), 8.89 (s, 1H, H-2 pyrid.), 9.03 (s, 1H, NH), ppm; ^13^C-NMR (DMSO-*d*_6_, 50 MHz) δ 35.20, 119.32, 120.27, 121.15, 124.83, 127.90, 128.87, 129.49, 130.91, 131.34, 132.13, 135.20, 138.62, 149.71, 150.11, 150.32, 151.49 ppm; anal. C 48.73, H 3.23, N 8.97% calcd for C_19_H_15_Cl_2_N_3_O_3_S_2_ C 48.17, H 3.00, N 8.85%.

*N-[(4-Chlorophenyl)carbamoyl]-4-[(5-methyl-1,3,4-thiadiazol-2-yl)thio]-3-pyridinesulfonamide* (**14**). Starting from 4-[(5-methyl-1,3,4-thiadiazol-2-yl)thio]-3-pyridinesulfonamide (**3**, 0.29 g), and 4-chloro- phenyl isocyanate (0.18 g), the title compound **14** was obtained (0.28 g, 63%): mp 167–171 °C; IR (KBr) ν_max_ 3297, 3201 (NH), 3076 (C_Ar_-H), 2924, 2855 (C-H), 1716 (C=O), 1610, 1547, 1492 (C=C, C=N), 1350, 1159 (SO_2_) cm^−1^; ^1^H-NMR (DMSO-*d*_6_, 200 MHz) δ 2.80 (s, 3H, CH_3_), 7.18 (d, 1H, H-5 pyrid.), 7.30–7.39 (m, 4H, Ph), 8.63 (d, 1H, H-6 pyrid.), 9.07 (s, 2H, H-2 pyrid. NH), 8.90 (s, 1H, NH), ppm; ^13^C-NMR (DMSO-*d*_6_, 125 MHz) δ 16.47, 121.36, 123.44, 127.60, 129.39, 133.28, 137.61, 145.94, 151.21, 153.96, 157.83, 172.56 ppm; anal. C 40.77, H 2.74, N 15.85% calcd for C_15_H_12_ClN_5_O_3_S_3_ C 40.65, H 2.60, N 16.08%.

*N-(Phenylcarbamoyl)-4-(4-phenylpiperazin-1-yl)-3-pyridinesulfonamide* (**15**). Starting from 4-(4-phenylpiperazin-1-yl)-3-pyridinesulfonamide (**4**, 0.32 g), and phenyl isocyanate (0.14 g), the title compound **15** was obtained (0.32 g, 73%): mp 154–156 °C; IR (KBr) ν_max_ 3466 (NH), 3059 (C_Ar_-H), 2826 (C-H), 1640 (C=O), 1599, 1524, 1504 (C=C, C=N), 1314 1138 (SO_2_) cm^−1^; ^1^H-NMR (DMSO-*d*_6_, 200 MHz) δ 3.33 (s, 4H, 2 × CH_2_), 3.82 (s, 4H, 2 × CH_2_), 6.77–7.06 (m, 4H, H_Ar_), 7.12–7.40 (m, 7H, H_Ar_, H-5 pyrid.), 8.39 (d, 1H, H-6 pyrid.), 8.77 (s, 1H, H-2 pyrid.), 8.92 (s, 1H, NH), 12.10 (br.s, 1H, NH), ppm; ^13^C-NMR (DMSO-*d*_6_, 50 MHz) δ 48.14, 51.06, 114.35, 115.68, 118.31, 119.34, 121.61, 128.72, 129.15, 129.29, 140.59, 150.82, 156,69 ppm; anal. C 60.39, H 5.30, N 16.01%. calcd for C_22_H_23_N_5_O_3_S, C 60.26, H 5.36, N 15.68%.

*N-[(4-Chlorophenyl)carbamoyl]-4-(4-phenylpiperazin-1-yl)-3-pyridinesulfonamide* (**16**). Starting from 4-(4-phenylpiperazin-1-yl)-3-pyridinesulfonamide (**4**, 0.32 g), and 4-chlorophenylisocyanate (0.18 g), the title compound **16** was obtained (0.27 g, 56%): mp 145–149 °C; IR (KBr) ν_max_ 3478, 3290 (NH), 3070 (C_Ar_-H), 2836 (C-H), 1641 (C=O), 1603, 1519, (C=C, C=N), 1320, 1133 (SO_2_) cm^−1^; ^1^H-NMR (DMSO-*d*_6_, 200 MHz) δ 3.30 (s, 4H, 2 × CH_2_), 3.96 (s, 4H, 2 × CH_2_), 6.78 (t, 1H, H-4 Ph), 6.94 (d, 2H, H-2,6 Ph), 7.14–7.31 (m, 5H, H_Ar_ and H-5 pyrid_._), 7.44 (d, 2H, H-2ʹ,6ʹ Ph), 8.32 (d, 1H, H-6 pyrid.), 8.89 (s, 1H, H-2 pyrid.), 8.95 (s, 1H, NH), 13.0 (br.s, 1H, NH), ppm; ^13^C-NMR (DMSO-*d*_6_, 125 MHz) δ 48.57, 51.11, 116.09, 119.81, 128.83, 129.53, 129.71, 151.12, 156.82 ppm; anal. C 55.99, H 4.70, N 14.84%. calcd for C_22_H_22_ClN_5_O_3_S, C 55.78, H 4,96, N 14.71%.

*N-[(3,4-Dichlorophenyl)carbamoyl]-4-(4-phenylpiperazin-1-yl)-3-pyridinesulfonamide* (**17**). Starting from 4-(4-phenylpiperazin-1-yl)-3-pyridinesulfonamide (**4**, 0.32 g), and 3,4-dichlorophenyl isocyanate (0.23 g), the title compound **17** was obtained (0.24 g, 47%): mp 172–175 °C; IR (KBr) ν_max_ 3425 (NH), 3062 (C_Ar_-H), 2919, 2855 (C-H), 1630 (C=O), 1599, 1577, 1540, 1506 (C=C, C=N), 1371, 1140 (SO_2_) cm^−1^; ^1^H-NMR (DMSO-*d*_6_, 200 MHz) δ 3.29 (s, 4H, 2 × CH_2_), 4.02 (s, 4H, 2 × CH_2_), 6.79 (t, 1H, H-4 Ph), 6.93 (d, 2H, H-2,6 Ph), 7.18–7.32 (m, 5H, H_Ar_ and H-5 pyrid.), 7.85 (s, 1H, H-2ʹ Ph), 8.27 (d, 1H, H-6 pyrid.), 8.88 (s, 1H, H-2 pyrid.), 9.12 (s, 1H, NH), 13.4 (br.s, 1H, NH), ppm; ^13^C-NMR (DMSO-*d*_6_, 125 MHz) δ 48.58, 50.89, 113.65, 116.10, 118.16, 118.95, 119.83, 121.83, 129.47, 129.71, 130.74, 131.24, 142.68, 146.01, 151.08, 156.64 ppm; anal. C 52.18, H 4.18, N 13.83%. calcd for C_22_H_21_Cl_2_N_5_O_3_S, C 51.78, H 4.05, N 13.86%.

*4-[4-(4-Chlorophenyl)piperazin-1-yl]-N-(phenylcarbamoyl)-3-pyridinesulfonamide* (**18**). Starting from 4-[4-(4-chlorophenyl)piperazin-1-yl]-3-pyridinesulfonamide (**4**, 0.35 g), and phenyl isocyanate (0.14 g), the title compound **18** was obtained (0.36 g, 76%): mp 177–181 °C; IR (KBr) ν_max_ 3404, 3278 (NH), 3083 (C_Ar_-H), 2923, 2855 (C-H), 1648 (C=O), 1650 1598, 1496 (C=C, C=N), 1311 1128 (SO_2_) cm^−1^; ^1^H-NMR (DMSO-*d*_6_, 200 MHz) δ 3.34 (s, 4H, 2 × CH_2_), 3.81 (s, 4H, 2 × CH_2_), 6.86 (t, 1H, H-4ʹ Ph), 6.98 (d, *J* = 8.9 Hz, 2H, H-2,6 Ph), 7.16 (t, 2H, H-3ʹ5ʹ Ph), 7.26 (d, *J* = 8.9 Hz, 2H, H-3,5 Ph), 7.31 (d, 1H H-5 pyrid.), 7.38 (d, 2H, H-2ʹ,6ʹ Ph), 8.40 (d, 1H, H-6 pyrid.), 8.77 (s, 1H, H-2 pyrid.), 8.92 (s, 1H, NH), ppm; ^13^C-NMR (DMSO-*d*_6_, 125 MHz) δ 48.32, 51.21, 117.52, 118.64, 123.22, 129.11, 129.37, 129.61, 150.02, 157.06 ppm; anal. C 55.99, H 4.70, N 14.84%. calcd for C_22_H_22_ClN_5_O_3_S, C 55.59, H 4.65, N 14.87%.

*N-[(4-Chlorophenyl)carbamoyl]-4-[4-(4-chlorophenyl)piperazin-1-yl]pyridine-3-sulfonamide* (**19**). Starting from 4-[4-(4-chlorophenyl)piperazin-1-yl]-3-pyridinesulfonamide (**5**, 0.35 g), and 4-chloro- phenyl isocyanate (0.18 g), the title compound **19** was obtained (0.28 g, 56%): mp 178–182 °C; IR (KBr) ν_max_ 3427, 3180 (NH), 3050 (C_Ar_-H), 2920, 2854 (C-H), 1642 (C=O), 1626, 1583, 1540, 1497 (C=C, C=N), 1278, 1153 (SO_2_) cm^−1^; ^1^H-NMR (DMSO-*d*_6_, 200 MHz) δ 3.32 (s, 4H, 2 × CH_2_), 3.95 (s, 4H, 2 × CH_2_), 6.96 (d, 2H, H-2,6 Ph), 7.17 (d, 2H, H-3,5 Phʹ), 7.23–7.31 (m, 3H, H-3,5 Ph and H-5 pyrid.), 7.44 (d, 2H, H-2,6 Phʹ), 8.33 (d, 1H, H-6 pyrid.), 8.90 (s, 1H, H-2 pyrid.), 8.93 (s, 1H, NH), ppm; anal. C 52.18, H 4.18, N 13.83%. calcd for C_22_H_21_Cl_2_N_5_O_3_S, C 52.38, H 3.98, N 13.71%.

*4-[4-(3,4-Dichlorophenyl)piperazin-1-yl]-N-(phenylcarbamoyl)-3-pyridinesulfonamide* (**20**). Starting from 4-[4-(3,4-dichlorophenyl)piperazin-1-yl]-3-pyridinesulfonamide (**6**, 0.39 g), and phenyl isocyanate (0.14 g), the title compound **20** was obtained (0.40 g, 80%): mp 177–180 °C; IR (KBr) ν_max_ 3443 (NH), 3058 (C_Ar_-H), 2837 (C-H), 1639 (C=O), 1595, 1526, 1510, 1485 (C=C, C=N), 1314, 1141 (SO_2_) cm^−1^; ^1^H-NMR (DMSO-*d*_6_, 200 MHz) δ 3.39 (s, 4H, 2 × CH_2_), 3.79 (s, 4H, 2 × CH_2_), 6.86 (t, 1H, H-4ʹ Ph), 6.96 (dd, *J_ortho_* = 8.2 Hz, *J_meta_* = 2.2 Hz, 1H, H-6 3,4-diClPh), 7.16 (m, 3H, H-5 3,4-diClPh and H-3,5 Ph), 7.30 (d,h 1H, H-5 pyrid.), 7.36–7.44 (m, 3H, H-2,6 Ph and H-2,3 4-diClPh), 8.40 (d, 1H, H-6 pyrid.), 8.77 (s, 1H, H-2 pyrid.), 8.91 (s, 1H, NH), ppm; anal. C 52.18, H 4.18, N 13.83%. calcd for C_22_H_21_Cl_2_N_5_O_3_S, C 52.51, H 4.11, N 13.81%.

*N-[(4-Chlorophenyl)carbamoyl]-4-[4-(3,4-dichlorophenyl)piperazin-1-yl]pyridine-3-sulfonamide* (**21**). Starting from 4-[4-(3,4-dichlorophenyl)piperazin-1-yl]-3-pyridinesulfonamide (**6**, 0.39 g), and 4-chlorophenyl isocyanate (0.18 g), the title compound **21** was obtained (0.35 g, 65%): mp 164–168 °C; IR (KBr) ν_max_ 3469 (NH), 3095 (C_Ar_-H), 2925, 2837 (C-H), 1641 (C=O), 1591, 1525, 1489 (C=C, C=N), 1306. 1141 (SO_2_) cm^−1^; ^1^H-NMR (DMSO-*d*_6_, 200 MHz) δ 3.38 (s, 4H, 2 × CH_2_), 3.93 (s, 4H, 2 × CH_2_), 6.94 (dd, 1H, H-6 3,4-diClPh), 7.17 (m, 3H, H-5 3,4-diClPh and H-3,5 Ph), 7.28 (d, 1H, H-5 pyrid.), 7.38–7.46 (m, 3H, H-2,6 Ph and H-2 3,4-diClPh), 8.33 (d, 1H, H-6 pyrid.), 8.89 (s, 1H, H-2 pyrid.), 8.92 (s, 1H, NH), ppm; ^13^C-NMR (DMSO-*d*_6_, 50 MHz) δ 47.32, 50.33, 113.71, 113.75, 115.44, 116.42, 119.47, 119.99, 124.43, 128.42, 129.28, 130.78, 131.82, 140.32, 150.41, 156.49 ppm. anal. C 48.86, H 3.73, N 12.95%. calcd. for C_22_H_20_Cl_3_N_5_O_3_S, C 48.82, H 3.70, N 12.81%.

*N-[(4-Chlorophenyl)carbamoyl]-4-[4-(2,5-dimethylphenyl)piperazin-1-yl]-3-pyridinesulfonamide* (**22**). Starting from 4-[4-(2,5-dimethylphenyl)piperazin-1-yl]-3-pyridinesulfonamide (**7**, 0.35 g), and 4-chlorophenyl isocyanate (0.18 g), the title compound **22** was obtained (0.36 g, 72%): mp 171–174 °C; IR (KBr) ν_max_ 3302 (NH), 3023 (C_Ar_-H), 2945, 2918, 2857 (C-H), 1642 (C=O), 1516 (C=C, C=N), 1320, 1129 (SO_2_) cm^−1^; ^1^H-NMR (DMSO-*d*_6_, 200 MHz) δ 2.24 (s, 6H, 2 × CH_3_), 2.96 (br.s, 4H, 2 × CH_2_), 3.93 (br.s, 4H, 2 × CH_2_), 6.78 (d, 1H, H-4 Ph), 6.86 (s. 1H, H-6 Ph), 7.06 (d, 1H, H-3 Ph), 7.17 (d, 2H, H-3,5 Ph) 7.30 (d, 1H, H-5 pyrid.), 7.47 (d, 2H, H-2,6 Ph), 8.33 (d, 1H, H-6 pyrid.), 8.89 (s, 1H, H-2 pyrid.), 8.97 (s, 1H, NH), ppm; ^13^C-NMR (DMSO-*d*_6_, 125 MHz) δ 17.84, 21.48, 51.83, 51.93, 114.17, 115.86, 119.66, 120.46, 124.45, 128.77, 129.24, 130.32, 131.33, 136.27, 141.13, 146.81, 151.24, 156.94, 157.63 ppm; anal. C 57.65, H 5.24, N 14.01%. calcd for C_24_H_26_ClN_5_O_3_S, C 57.21, H 5.44, N 14.07%.

*N-[(4-Chlorophenyl)carbamoyl]-4-[4-(pyridin-2-yl)piperazin-1-yl]-3-pyridinesulfonamide* (**23**). Starting from 4-[4-(pyridin-2-yl)piperazin-1-yl]-3-pyridinesulfonamide (**8**, 0.32 g), and 4-chlorophenyl isocyanate (0.18 g), the title compound **23** was obtained (0.30 g, 64%): mp 187–193 °C; IR (KBr) ν_max_ 3307 (NH), 3073 (C_Ar_-H), 2923, 2854 (C-H), 1638 (C=O), 1609, 1593, 1505, (C=C, C=N), 1315, 1149 (SO_2_) cm^−1^; ^1^H-NMR (DMSO-*d*_6_, 200 MHz) δ 3.67 (s, 4H, 2 × CH_2_), 3.76 (s, 4H, 2 × CH_2_), 6.66 (t, 1H, H-5′ pyrid.), 6.85 (d, 1H, H-3′ pyrid.), 7.13 (m, 3H, H-3,5 Ph, H-5 pyrid), 7.46 (d, 2H, H-2,6 Ph), 7.56 (t, 1H, H-4′ pyrid.), 8.13 (d, 1H, H-6′ pyrid.), 8.29 (d, 1H, H-6 pyrid.), 8.81 (s, 1H, H-2 pyrid.), 8.91 (s, 1H, NH), ppm; ^13^C-NMR (DMSO-*d*_6_, 125 MHz) δ 45.01, 50.73, 107.79, 113.70, 113.76, 119.61, 124.16, 128.70, 130.79, 138.30, 141.43, 145.69, 148.25, 149.24, 156.08, 158.07, 159.38 ppm; anal. C 53.33, H 4.48, N 17.77%. calcd for C_21_H_21_ClN_6_O_3_S, C 52.44, H 4.19, N 17.67%.

*N-(Phenylcarbamoyl)-4-(3,4,5-trimethyl-1H-pyrazol-1-yl)-3-pyridinesulfonamide* (**24**). Starting from 4-(3,4,5-trimethyl-1*H*-pyrazol-1-yl)-3-pyridinesulfonamide (**9**, 0.27 g), and phenyl isocyanate (0.14 g), the title compound **24** was obtained (0.33 g, 87%): mp 161–164 °C; IR (KBr) ν_max_ 3254, 3199 (NH), 3079, 3018 (C_Ar_-H), 2927, 2860 (C-H), 1730 (C=O), 1583, 1499 (C=C, C=N), 1363, 1350, 1170 (SO_2_) cm^−1^; ^1^H-NMR (DMSO-*d*_6_, 200 MHz) δ 1.93 (s, 3H, CH_3_), 2.05 (s, 3H, CH_3_), 2.17 (s, 3H, CH_3_), 7.04 (t, 1H, H-4 Ph), 7.28 (m, 4H, Ph), 7.57 (d, *J* = 5.2 Hz, 1H H-5 pyrid.), 8.96 (d, *J* = 5.2 Hz, 1H, H-6 pyrid.), 9.08 (s, 1H, H-2 pyrid.), 9.27 (s, 1H, NH), 9.7 (br.s, 1H, NH), ppm; ^13^C-NMR (DMSO-*d*_6_, 125 MHz) δ 8.56, 10.61, 12.64, 114.15, 119.43, 124.07, 124.21, 129.65, 132.08, 138.48, 144.97, 149.67, 150.12, 152.88, 155.93 ppm; anal. C 56.09, H 4.97, N 18.17%. calcd for C_18_H_19_N_5_O_3_S, C 55.78, H 4.89, N 18.18%.

*N-[(4-Chlorophenyl)carbamoyl]-4-(3,4,5-trimethyl-1H-pyrazol-1-yl)-3-pyridinesulfonamide* (**25**). Starting from 4-(3,4,5-trimethyl-1*H*-pyrazol-1-yl)-3-pyridinesulfonamide (**9**, 0.27 g), and 4-chloro- phenyl isocyanate (0.18 g), the title compound **25** was obtained (0.31 g, 75%): mp 178–182 °C; IR (KBr) ν_max_ 3247, 3186 (NH), 3010, 3035 (C_Ar_-H), 2992, 2862 (C-H), 1727 (C=O), 1588, 1552, 1496 (C=C, C=N), 1384, 1363, 1170 (SO_2_) cm^−1^; ^1^H-NMR (DMSO-*d*_6_, 200 MHz) δ 1.93 (s, 3H, CH_3_), 2.05 (s, 3H, CH_3_), 2.16 (s, 3H, CH_3_), 7.35 (s, 4H, Ph), 7.57 (d, 1H H-5 pyrid.), 8.96 (d, 1H, H-6 pyrid.), 9.21 (s, 1H, H-2 pyrid.), 9.26 (s, 1H, NH), ppm; ^13^C-NMR (DMSO-*d*_6_, 50 MHz) δ 8.13, 10.19, 12.21, 113.75, 120.69, 123.83, 127.29, 129.11, 131.64, 137.07, 138.09, 144.56, 149.28, 149.72, 152.40, 155.57 ppm; anal. C 51.49, H 4.32, N 16.68%. calcd for C_18_H_18_ClN_5_O_3_S, C 51.22, H 4.20, N 16.57%.

*N-[(3,4-Dichlorophenyl)carbamoyl]-4-(3,4,5-trimethyl-1H-pyrazol-1-yl)-3-pyridinesulfonamide* (**26**). Starting from 4-(3,4,5-trimethyl-1*H*-pyrazol-1-yl)-3-pyridinesulfonamide (**9**, 0.27 g), and 3,4-dichloro-phenyl isocyanate (0.23 g), the title compound **26** was obtained (0.25 g, 55%): mp 163–166 °C; IR (KBr) ν_max_ 3324 (NH), 3104 (C_Ar_-H), 2924, 2856 (C-H), 1727 (C=O), 1599, 1581, 1477 (C=C, C=N), 1381 1169 (SO_2_) cm^−1^; ^1^H-NMR (DMSO-*d*_6_, 200 MHz) δ 1.92 (s, 3H, CH_3_), 2.04 (s, 3H, CH_3_), 2.14 (s, 3H, CH_3_), 7.25 (dd, 1H, H-6 Ph), 7.55 (m, 2H, H-5 Ph, H-5 pyrid.), 7.69 (d, 1H H-2 Ph), 8.96 (d, 1H, H-6 pyrid.), 9.26 (s, 1H, H-2 pyrid.), 9.40 (s, 1H, NH), ppm; ^13^C-NMR (DMSO-*d*_6_, 125 MHz) δ 8.55, 10.60, 12.64, 114.16, 119.69, 120.63, 124.29, 125.50, 131.48, 131.85, 132.12, 138.51, 138.78, 144.96, 149.97, 150.13, 152.75, 156.00 ppm; anal. C 47.58, H 3.77, N 15.41% calcd for C_18_H_17_Cl_2_N_5_O_3_S, C 47.45, H 3.81, N 15.38%.

*4-(4-Butyl-3,5-dimethyl-1H-pyrazol-1-yl)-N-[(4-chlorophenyl)carbamoyl]-3-pyridinesulfonamide* (**27**). Starting from 4-(4-butyl-3,5-dimethyl-1*H*-pyrazol-1-yl)-3-pyridinesulfonamide (**10**, 0.31 g), and 4-chlorophenyl isocyanate (0.18 g), the title compound **27** was obtained (0.35 g, 76%): m.p. (EtOAc/Et_2_O). 138–142 °C ; IR (KBr) ν_max_ 3344 (NH), 3127, (C_Ar_-H), 2947, 2865 (C-H), 1726 (C=O), 1602, 1579, 1541, 1495 (C=C, C=N), 1385, 1164 (SO_2_) cm^−1^; ^1^H-NMR (DMSO-*d*_6_, 200 MHz) δ 0.90 (t, 3H, CH_3_), 1.36 (m, 4H, 2 × CH_2_), 2.07 (s, 3H, CH_3_), 2.19 (s, 3H, CH_3_), 2.36 (t, 2H, CH_2_), 7.36 (s, 4H, Ph), 7.60 (d, 1H H-5 pyrid.), 8.96 (d, 1H, H-6 pyrid.), 9.25 (s, 1H, H-2 pyrid.), 9.27 (s, 1H, NH), ppm; ^13^C-NMR (DMSO-*d*_6_, 125 MHz) δ 10.61, 12.68, 14.55, 22.46, 23.22, 32.85, 119.09, 121.04, 124.19, 127.66, 129.51, 132.13, 137.50, 138.48, 144.91, 149.77, 149.79, 152.80, 155.88 ppm; anal. C 54.60, H 5.24, N 15.16% calcd for C_21_H_24_ClN_5_O_3_S, C 54.21, H 5.16, N 14.99%.

### 3.3. In Vitro Anticancer Screening

Antitumor evaluation of compounds **11**, **12**, **14**–**21** and **24**–**26** was performed at the National Cancer Institute according to NCI-60 DTP Human Tumor Cell Line Screen procedure [25,26,27,28,29].

## 4. Conclusions 

We have obtained a novel series of 4-substituted *N*-(R^2^-phenylcarbamoyl)-3-pyridinesulfonamide derivatives **11**–**27** by the reaction of 4-substituted pyridine-3-sulfonamides **2**–**10** with aryl isocyanates in the presence of potassium carbonate. The compounds **11**, **12**, **14**–**21** and **24**–**26** have been screened *in vitro* for their anticancer activity at the U.S. National Cancer Institute. We found that many of the investigated compounds exhibited structure-dependent moderate or weak anticancer activity. Considering the structure-activity relationships, we conclude that in general the presence of a 4-chlorophenylcarbamoyl moiety attached to the sulfonamide functionality exerts in most cases a favorable influence on anticancer activity and selectivity of the tested diarylsulfonylurea derivatives **12**, **14**, **16**, **19**, **21** and **25**. Thus, the most active compound, *N*-[(4-chlorophenyl)carbamoyl]-4-[4-(3,4-dichlorophenyl)piperazin-1-yl]pyridine-3-sulfonamide (**21**) exhibited a good activity profile and selectivity toward the subpanels of either leukemia and colon cancer, or melanoma, with average GI_50_ values ranging from 13.6 to 14.9 µM, and could be considered as a lead compound for further optimization.

## Supplementary Materials

Supplementary materials can be accessed at: http://www.mdpi.com/1420-3049/20/07/12029/s1.

## References

[1] Ceras J., Cirauqui N., Pérez-Silanes S., Aldana I., Monge A., Galiano S. (2012). Novel Sulfonylurea Derivatives as H_3_ Receptor Antagonists. Preliminary SAR Studies. Eur. J. Med. Chem..

[2] Khatri N., Madan K. (2014). Models for H_3_ Receptor Antagonist Activity of Sulfonylurea Derivatives. J. Mol. Graph. Model..

[3] Michaux C., Dogné J.M., Rolin S., Masereel B., Wouters J., Durant F. (2003). A Pharmacophore Model for Sulphonyl-Urea (-Cyanoguanidine) Compounds with Dual Action, Thromboxane Receptor Antagonists and Thromboxane Synthase Inhibitors. Eur. J. Med. Chem..

[4] Bambi-Nyanguile S.M., Hanson J., Ooms A., Alpan L., Kolh P., Dogné J.M., Pirotte B. (2013). Synthesis and Pharmacological Evaluation of 2-Aryloxy/arylamino-5-Cyanobenzenesulfonylureas as Novel Thromboxane A2 Receptor Antagonists. Eur. J. Med. Chem..

[5] Pan L., Jiang Y., Liu Z., Liu X.H., Liu Z., Wang G., Li Z.M., Wang D. (2012). Synthesis and Evaluation of Novel Monosubstituted Sulfonylurea Derivatives as Antituberculosis Agents. Eur. J. Med. Chem..

[6] León C., Rodrigues J., Gamboa de Domínguez N., Charris J., Gut J., Rosenthal P.J., Domínguez J.N. (2007). Synthesis and Evaluation of Sulfonylurea Derivatives as Novel Antimalarials. Eur. J. Med. Chem..

[7] Lee Y.T., Cui C.J., Chow E.W.L., Pue N., Lonhienne T., Wang J.G., Fraser J.A., Guddat L.W. (2013). Sulfonylureas Have Antifungal Activity and Are Potent Inhibitors of Candida Albicans Acetohydroxyacid Synthase. J. Med. Chem..

[8] El-Deeb I.M., Bayoumi S.M., El-Sherbeny M.A., Abdel-Aziz A.A.M. (2010). Synthesis and Antitumor Evaluation of Novel Cyclic Arylsulfonylureas: ADME-T and Pharmacophore Prediction. Eur. J. Med. Chem..

[9] El-Sherbeny M., Abdel-Aziz A.M., Ahmed M. (2010). Synthesis and Antitumor Evaluation of Novel Diarylsulfonylurea Derivatives: Molecular Modeling Applications. Eur. J. Med. Chem..

[10] Avupati V.R., Yejella R.P., Guntuku G., Gunta P. (2012). Synthesis, Characterization and *in Vitro* Biological Evaluation of Some Novel Diarylsulfonylureas as Potential Cytotoxic and Antimicrobial Agents. Bioorg. Med. Chem. Lett..

[11] Rathore P., Yaseen S., Ovais S., Bashir R., Yaseen R., Hameed A.D., Samim M., Gupta R., Hussain F., Javed K. (2014). Synthesis and Evaluation of Some New Pyrazoline Substituted Benzenesulfonylureas as Potential Antiproliferative Agents. Bioorg. Med. Chem. Lett..

[12] Zhang Z.J., Tian J., Wang L.T., Wang M.J., Nan X., Yang L., Liu Y.Q., Morris-Natschke S.L., Lee K.H. (2014). Design, Synthesis and Cytotoxic Activity of Novel Sulfonylurea Derivatives of Podophyllotoxin. Bioorg. Med. Chem..

[13] Howbert J., Grossman C.S., Crowell T., Rieder B.J., Harper R.W., Kramer K.E., Tao E.V., Aikins J., Poore G., Rinzel S.M. (1990). Novel Agents Effective against Solid Tumors: The Diarylsulfonylureas. Synthesis, Activities, and Analysis of Quantitative Structure-Activity Relationships. J. Med. Chem..

[14] Scozzafava A., Owa T., Mastrolorenzo A., Supuran C.T. (2003). Anticancer and Antiviral Sulfonamides. Curr. Med. Chem..

[15] Pasello G., Urso L., Conte P., Favaretto A. (2013). Effects of Sulfonylureas on Tumor Growth: A Review of the Literature. Oncologist.

[16] Brzozowski Z., Sławiński J., Sączewski F., Innocenti A., Supuran C.T. (2010). Carbonic anhydrase inhibitors: Synthesis and inhibition of the human cytosolic isozymes I and II and transmembrane isozymes IX, XII (cancer-associated) and XIV with 4-substituted 3-pyridinesulfonamides. Eur. J. Med. Chem..

[17] Sławiński J., Szafrański K., Vullo D., Supuran C.T. (2013). Carbonic Anhydrase Inhibitors. Synthesis of Heterocyclic 4-Substituted Pyridine-3-Sulfonamide Derivatives and Their Inhibition of the Human Cytosolic Isozymes I and II and Transmembrane Tumor-Associated Isozymes IX and XII. Eur. J. Med. Chem..

[18] Pacchiano F., Carta F., McDonald P.C., Lou Y., Vullo D., Scozzafava A., Dedhar S., Supuran C.T. (2011). Ureido-Substituted Benzenesulfonamides Potently Inhibit Carbonic Anhydrase IX and Show Antimetastatic Activity in a Model of Breast Cancer Metastasis. J. Med. Chem..

[19] Lou Y., McDonald P.C., Oloumi A., Chia S., Ostlund C., Ahmadi A., Kyle A., Auf Dem Keller U., Leung S., Huntsman D. (2011). Targeting Tumor Hypoxia: Suppression of Breast Tumor Growth and Metastasis by Novel Carbonic Anhydrase IX Inhibitors. Cancer Res..

[20] Pastorekova S., Zatovicova M., Pastorek J. (2008). Cancer-Associated Carbonic Anhydrases and Their Inhibition. Curr. Pharm. Des..

[21] Supuran C.T. (2008). Carbonic Anhydrases: Novel Therapeutic Applications for Inhibitors and Activators. Nat. Rev. Drug Discov..

[22] Neri D., Supuran C.T. (2011). Interfering with pH Regulation in Tumours as a Therapeutic Strategy. Nat. Rev. Drug Discov..

[23] Supuran C.T. (2012). Inhibition of Carbonic Anhydrase IX as a Novel Anticancer Mechanism. World J. Clin. Oncol..

[24] Gieling R.G., Williams K.J. (2012). Carbonic Anhydrase IX as a Target for Metastatic Disease. Bioorg. Med. Chem..

[25] Alley M.C., Scudiero D.A., Monks P.A., Hursey M.L., Czerwinski M.J., Fine D.L., Abbott B.J., Mayo J.G., Shoemaker R.H., Boyd M.R. (1988). Feasibility of Drug Screening with Panels of Human Tumor Cell Lines Using a Microculture Tetrazolium Assay. Cancer Res..

[26] Grever M.R., Schepartz S.A., Chabner B.A. (1992). The National Cancer Institute: Cancer Drug Discovery and Development Program. Semin. Oncol..

[27] Boyd M.R., Paull K.D. (1995). Some Practical Considerations and Applications of the National Cancer Institute *in Vitro* Anticancer Drug Discovery Screen. Drug Dev. Res..

[28] Shoemaker R.H. (2006). The NCI60 Human Tumour Cell line Anticancer Drug Screen. Nat. Rev..

[29] NCI-60 DTP Human Tumor Cell Line Screen. http://dtp.nci.nih.gov/branches/btb/ivclsp.html.

[30] Cancer Screening Data. http://dtp.nci.nih.gov/dtpstandard/cancerscreeningdata/index.jsp.

