# Supplementary material for: Synthesis of Novel 1-(4-Substituted pyridine-3-sulfonyl)-3-phenylureas with Potential Anticancer Activity
Source: Molecules. 2015 Jul 1;20(7):12029–44. doi: 10.3390/molecules200712029 (PMC6332147; doi:10.3390/molecules200712029)
Supplement: Supplementary file 1 [file molecules-20-12029-s001.pdf]

Supplementary Materials

Synthesis of Novel 1-(4-Substituted pyridine-3-sulfonyl)-3-phenylureas with Potential Anticancer Activity

Table of Contents

Table S1. Inhibition growth percent (IGP [%]) for tested compounds (**11**, **12**, **14-21**, **24-26**) against all NCI-60 cancer cell lines at single concentration 10^{-5} M, and anticancer *in vitro* data for compound **21**. S2

Spectrum S1. ^{1}H-NMR of compound **12** (200 MHz, DMSO-d_6). S4
Spectrum S2. ^{13}C-NMR of compound **12** (125 MHz, DMSO-d_6). S5

Spectrum S3. ^{1}H-NMR of compound **13** (200 MHz, DMSO-d_6). S6
Spectrum S4. ^{13}C-NMR of compound **13** (50 MHz, DMSO-d_6). S7

Spectrum S5. ^{1}H-NMR of compound **15** (200 MHz, DMSO-d_6). S8
Spectrum S6. ^{13}C-NMR of compound **15** (50 MHz, DMSO-d_6). S9

Spectrum S7. ^{1}H-NMR of compound **17** (200 MHz, DMSO-d_6). S10
Spectrum S8. ^{13}C-NMR of compound **17** (125 MHz, DMSO-d_6). S11

Spectrum S9. ^{1}H-NMR of compound **21** (200 MHz, DMSO-d_6). S12
Spectrum S10. ^{13}C-NMR of compound **21** (50 MHz, DMSO-d_6). S13

Spectrum S11. ^{1}H-NMR of compound **24** (200 MHz, DMSO-d_6). S14
Spectrum S12. ^{13}C-NMR of compound **24** (125 MHz, DMSO-d_6). S15

Spectrum S13. ^{1}H-NMR of compound **25** (200 MHz, DMSO-d_6). S16
Spectrum S14. ^{13}C-NMR of compound **25** (50 MHz, DMSO-d_6). S17

Spectrum S15. ^{1}H-NMR of compound **26** (200 MHz, DMSO-d_6). S18
Spectrum S16. ^{13}C-NMR of compound **26** (125 MHz, DMSO-d_6). S19

Graph S1. NCI Cancer Screen Current Data—DTP 60 cell/5 dose for Sulofenur (NSC-642684)—GI_{50} [log10(M)]. S20

Graph S2. NCI Cancer Screen Current Data—DTP 60 cell/5 dose for Sulofenur (NSC-642684)—TGI [log10(M)]. S21

Graph S3. NCI Cancer Screen Current Data—DTP 60 cell/5 dose for Sulofenur (NSC-642684)—LC_{50} [log10(M)]. S22

References S23

Table S1. Inhibition growth percent (IGP [%]) for tested compounds (**11**, **12**, **14**–**21**, **24**–**26**) against NCI-60 cancer cell lines at single concentration 10^{-5} M, and anticancer *in vitro* data for compound **21** (right three columns: GI_{50}, TGI and LC_{50} [μM]) [a].

	IGP [%] of Compound													GI_{50} [b]	TGI [c]	LC_{50} [d]
**Panel/Cell Line**	**11**	**12**	**14**	**15**	**16**	**17**	**18**	**19**	**20**	**21**	**24**	**25**	**26**		**21**	
Leukemia																
CCRF-CEM	1	4	27	30	7	NT	2	11	*	85	27	19	NT	8.2	43.6	>100
HL-60(TB)	3	17	NT	26	NT	*	4	9	8	120	19	22	*	24	69.7	>100
K-562	6	67	NT	45	NT	7	12	70	20	95	42	72	*	3	>100	>100
MOLT-4	7	21	NT	27	NT	6	5	18	9	85	9	20	8	19.9	68.1	>100
RPMI-8226	*	*	NT	70	NT	*	1	7	*	93	72	10	*	14.7	54.2	>100
SR	NT	15	NT	16	NT	12	NT	NT	NT	95	1	29	19	11.8	51.6	>100
NSCLC																
A-549/ATCC	*	16	13	16	2	*	2	14	0	94	NT	17	*	14.7	62.8	>100
EKVX	NT	NT	11	NT	6	NT	NT	NT	NT	NT	NT	*	NT	NT	NT	NT
HOP-62	*	*	6	10	10	*	1	1	*	82	7	*	*	18.8	43.5	>100
HOP-92	*	*	NT	53	NT	*	*	2	*	131	28	*	NT	NT	NT	NT
NCI-H226	4	*	*	13	*	*	4	1	10	84	6	*	*	13.4	57.9	>100
NCI-H23	*	*	*	8	*	*	*	*	*	98	6	5	*	19.9	51.9	>100
NCI-H322M	5	14	2	8	6	11	2	8	2	123	0	1	*	18.3	>100	>100
NCI-H460	*	3	10	17	*	*	*	2	*	93	17	5	*	17.2	69.4	>100
NCI-H522	7	25	*	43	*	8	9	19	5	110	28	12	8	14.1	49.4	>100
Colon cancer																
COLO 205	*	*	*	15	*	*	*	*	*	157	16	*	*	16.8	33	65.0
HCC-2998	11	1	*	1	*	*	11	9	2	73	*	2	*	15.5	31.6	64.3
HCT-116	1	9	27	21	17	*	*	17	6	92	21	19	*	15.1	34.4	78.2
HCT-15	*	39	37	*	31	9	*	28	*	41	*	53	4	6.4	68.3	>100
HT29	*	8	*	32	*	*	*	7	*	104	19	8	*	15.9	45.7	>100
KM12	*	43	*	25	4	7	NT	24	14	89	27	49	*	13	>100	>100
SW-620	*	24	30	10	21	*	*	30	*	82	12	40	*	14.4	>100	>100
CNS cancer																
SF-268	*	18	3	1	6	*	*	19	6	71	*	26	*	23.6	>100	>100
SF-295	11	13	6	43	13	10	*	10	NT	147	48	*	1	12.2	37.9	>100
SF-539	*	12	*	*	8	4	5	3	1	65	3	5	*	13.1	29	64.4
SNB-19	*	11	*	14	0	*	1	*	*	81	7	5	0	19.1	57.6	>100
SNB-75	16	8	12	11	8	NT	24	14	22	81	5	12	NT	25.6	72.1	>100
U251	4	4	11	34	7	*	7	10	3	93	NT	2	*	13	36.5	>100
Melanoma																
LOX IMVI	7	NT	59	10	56	NT	3	47	4	96	10	59	NT	NT	NT	NT
MALME-3M	9	1	*	58	*	3	6	6	5	101	44	2	*	20.3	61.9	>100
M14	*	17	16	27	17	*	*	9	0	77	25	29	*	8.9	53.4	>100

Table S1. *Cont.*

	IGP [%] of Compound													GI$_{50}$ [b]	TGI [c]	LC$_{50}$ [d]
**Panel/Cell Line**	**11**	**12**	**14**	**15**	**16**	**17**	**18**	**19**	**20**	**21**	**24**	**25**	**26**		**21**	
MDA-MB-435	*	9	16	11	12	*	*	*	*	93	15	23	*	18.9	>100	>100
SK-MEL-2	NT	2	*	14	*	*	*	*	*	114	4	*	*	NT	NT	NT
SK-MEL-28	*	1	*	15	*	*	*	*	*	41	14	*	4	20.7	43.1	89.9
SK-MEL-5	*	21	15	31	15	4	*	12	2	141	27	15	4	10.9	36.7	>100
UACC-257	*	*	3	20	*	*	2	3	*	122	NT	2	*	23.1	79.8	>100
UACC-62	*	57	51	33	38	*	*	32	*	73	31	42	10	1.5	15.5	>100
Ovarian cancer																
IGROV1	2	16	*	13	*	8	*	7	*	104	11	1	*	16.3	68.1	>100
OVCAR-3	*	*	*	42	*	*	*	*	*	75	50	*	*	16.6	36.7	81.1
OVCAR-4	*	*	9	27	*	*	1	*	*	82	17	*	*	22.5	90.8	>100
OVCAR-5	*	29	21	2	10	1	*	*	*	69	3	29	*	26.4	>100	>100
OVCAR-8	2	3	*	29	*	*	4	*	1	89	NT	5	*	18.6	>100	>100
NCI/ADR-RES	*	10	12	*	9	*	*	15	*	31	*	24	*	18.4	>100	>100
SK-OV-3	2	*	0	6	4	*	*	*	*	105	*	*	4	35.9	97.2	>100
Renal cancer																
786-0	*	2	1	6	2	*	2	9	4	76	3	12	*	16.1	43.7	>100
A498	15	19	33	4	42	5	17	13	12	80	7	6	0	15.2	63.1	>100
ACHN	*	31	22	25	21	*	*	24	*	93	26	25	*	11.6	>100	>100
CAKI-1	2	33	29	*	28	11	*	23	*	65	5	17	2	6.3	>100	>100
RXF-393	*	*	*	1	*	*	*	*	*	62	*	*	*	20.6	55.1	>100
SN12C	*	15	1	21	3	*	*	*	1	92	19	*	*	18.4	>100	>100
TK-10	9	4	*	13	*	*	6	7	8	72	0	2	*	33.1	>100	>100
UO-31	12	18	17	27	19	9	5	21	10	110	20	29	16	9.8	27.3	75.0
Prostate cancer																
PC-3	11	6	14	54	17	*	6	21	16	101	51	12	*	14.4	71.1	>100
DU-145	*	16	5	*	7	*	*	18	*	67	1	32	*	17.3	55.7	>100
Breast cancer																
MCF7	3	5	5	28	5	3	5	7	9	88	18	*	6	19.7	97.4	>100
MDA-MB-231/ATCC	*	9	1	*	3	*	6	*	*	100	*	*	*	14.6	63.3	>100
HS578T	*	2	9	NT	*	1	*	*	*	64	12	6	*	24	98.1	>100
BT-549	*	16	16	2	14	*	8	12	6	85	4	23	*	16.1	44.7	>100
T-47D	2	20	21	33	17	6	10	27	9	92	26	18	7	13.9	87.6	>100
MDA-MB-468	*	7	NT	59	NT	3	*	*	*	109	57	*	*	18.9	>100	>100

[a] Data obtained from NCI-60 DTP human tumor cell line screening [1–5]; [b] GI$_{50}$: molar concentration that inhibits 50% net cell growth; [c] TGI: molar concentration giving total growth inhibition; [d] LC$_{50}$: molar concentration causing 50% net cell death; NT – not tested; * IGP ≤ 0%.

No	ppm	Hz	intensity
1	2.493	498.55	63.910
2	4.504	900.66	88.320
3	7.235	1446.72	76.597
4	7.259	1451.57	50.603
5	7.330	1465.85	171.841
6	7.383	1476.47	11.791
7	7.444	1488.56	47.773
8	7.475	1494.79	34.321
9	7.634	1526.66	27.575
10	7.662	1532.15	28.288
11	8.572	1714.20	22.083
12	8.598	1719.32	20.122
13	8.854	1770.61	45.741
14	8.900	1779.76	36.608

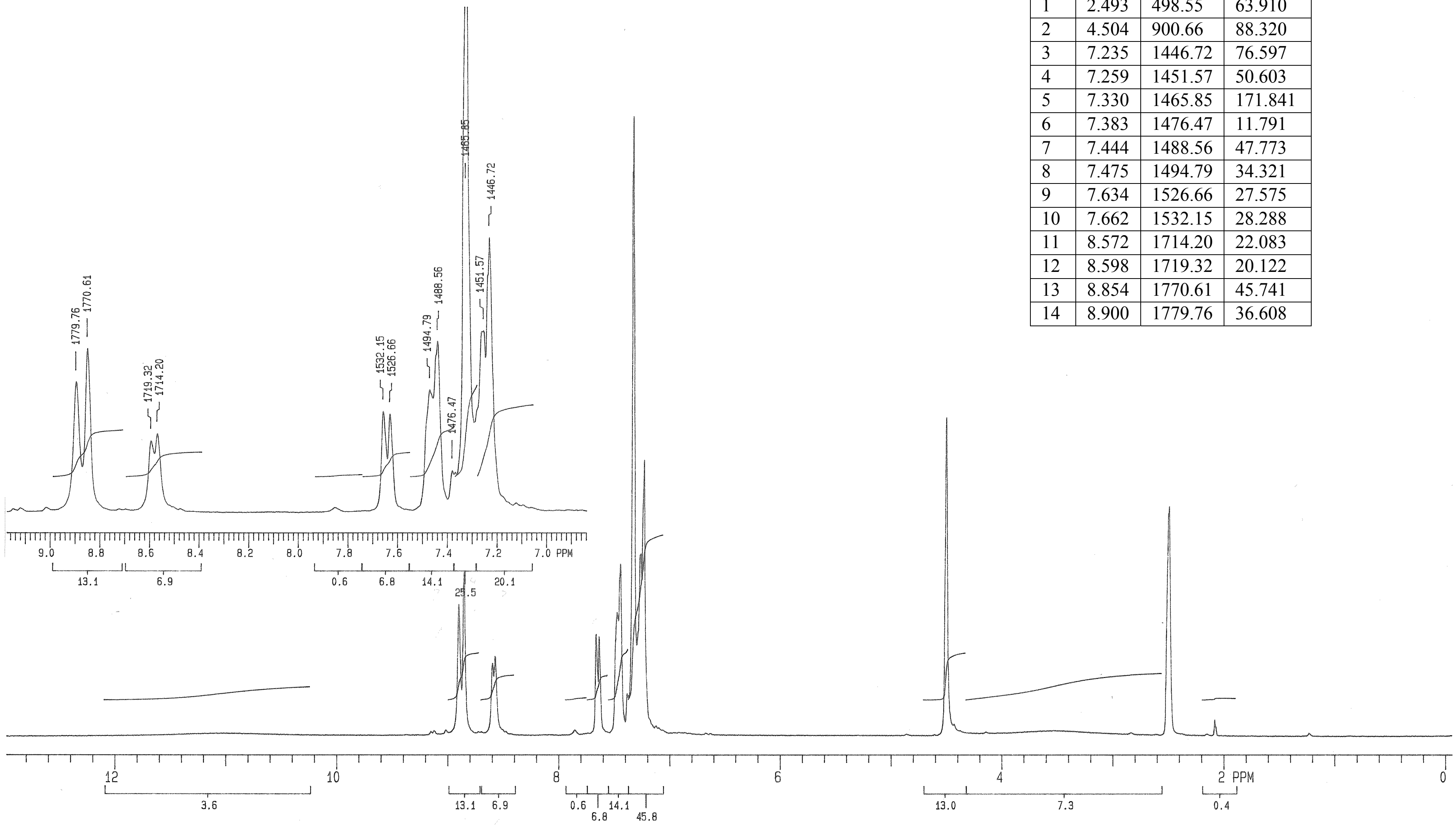

Spectrum S1. ^{1}H-NMR of compound **12** (200 MHz, DMSO-d_6).

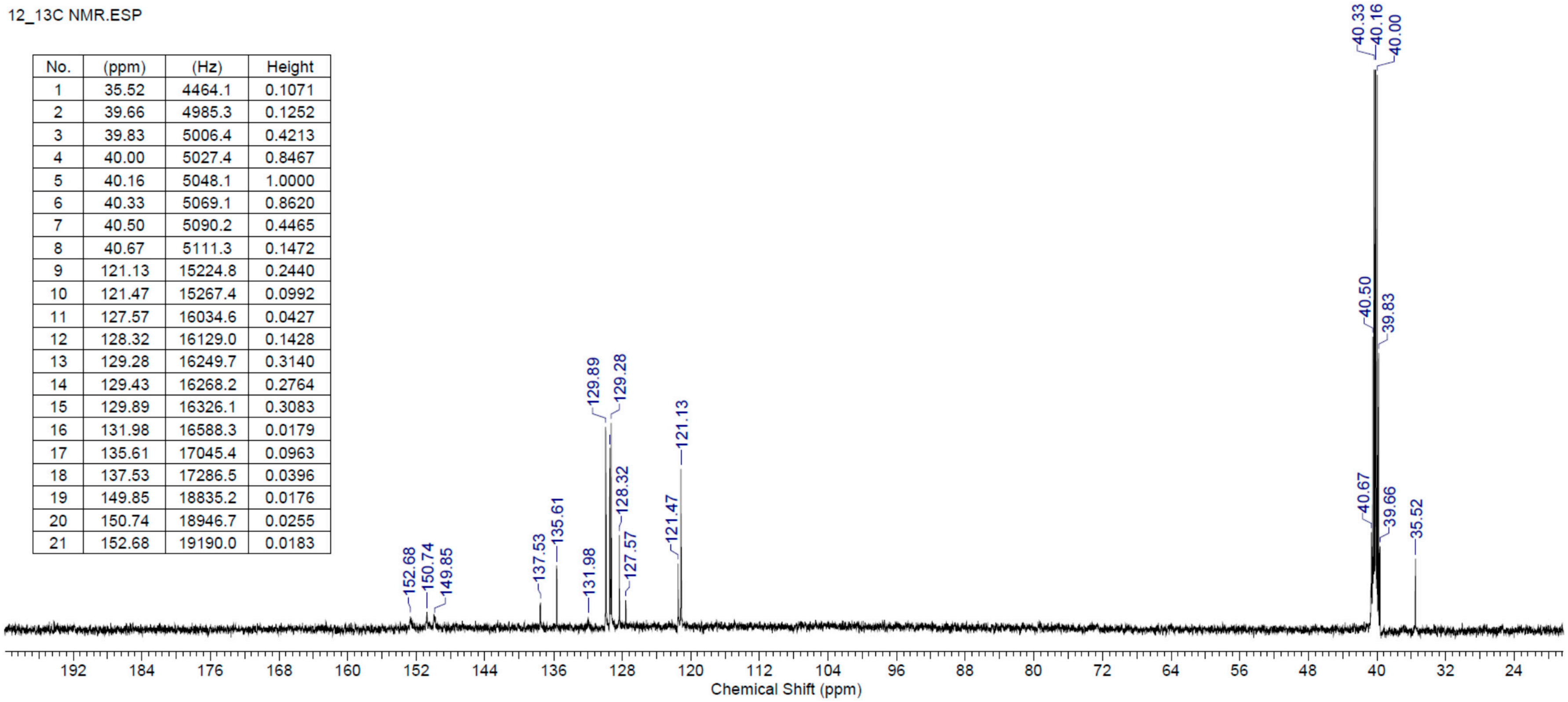

No.	(ppm)	(Hz)	Height
1	35.52	4464.1	0.1071
2	39.66	4985.3	0.1252
3	39.83	5006.4	0.4213
4	40.00	5027.4	0.8467
5	40.16	5048.1	1.0000
6	40.33	5069.1	0.8620
7	40.50	5090.2	0.4465
8	40.67	5111.3	0.1472
9	121.13	15224.8	0.2440
10	121.47	15267.4	0.0992
11	127.57	16034.6	0.0427
12	128.32	16129.0	0.1428
13	129.28	16249.7	0.3140
14	129.43	16268.2	0.2764
15	129.89	16326.1	0.3083
16	131.98	16588.3	0.0179
17	135.61	17045.4	0.0963
18	137.53	17286.5	0.0396
19	149.85	18835.2	0.0176
20	150.74	18946.7	0.0255
21	152.68	19190.0	0.0183

Spectrum S2. ^{13}C-NMR of compound **12** (125 MHz, DMSO-d_6).

No	ppm	Hz	Intensity
1	2.487	497.36	106.003
2	2.495	498.93	141.362
3	4.432	886.35	12.498
4	4.501	900.03	158.268
5	7.236	1446.99	124.105
6	7.243	1448.40	149.125
7	7.254	1450.65	97.250
8	7.270	1453.83	95.731
9	7.286	1456.94	76.366
10	7.298	1459.33	72.261
11	7.361	1472.03	8.863
12	7.437	1487.22	68.059
13	7.444	1488.69	78.645
14	7.479	1495.71	129.546
15	7.523	1504.37	60.987
16	7.659	1531.66	59.410
17	7.676	1535.02	95.860
18	7.687	1537.27	124.955
19	8.576	1715.02	38.927
20	8.603	1720.39	37.832
21	8.896	1778.99	64.896
22	9.032	1806.14	76.257

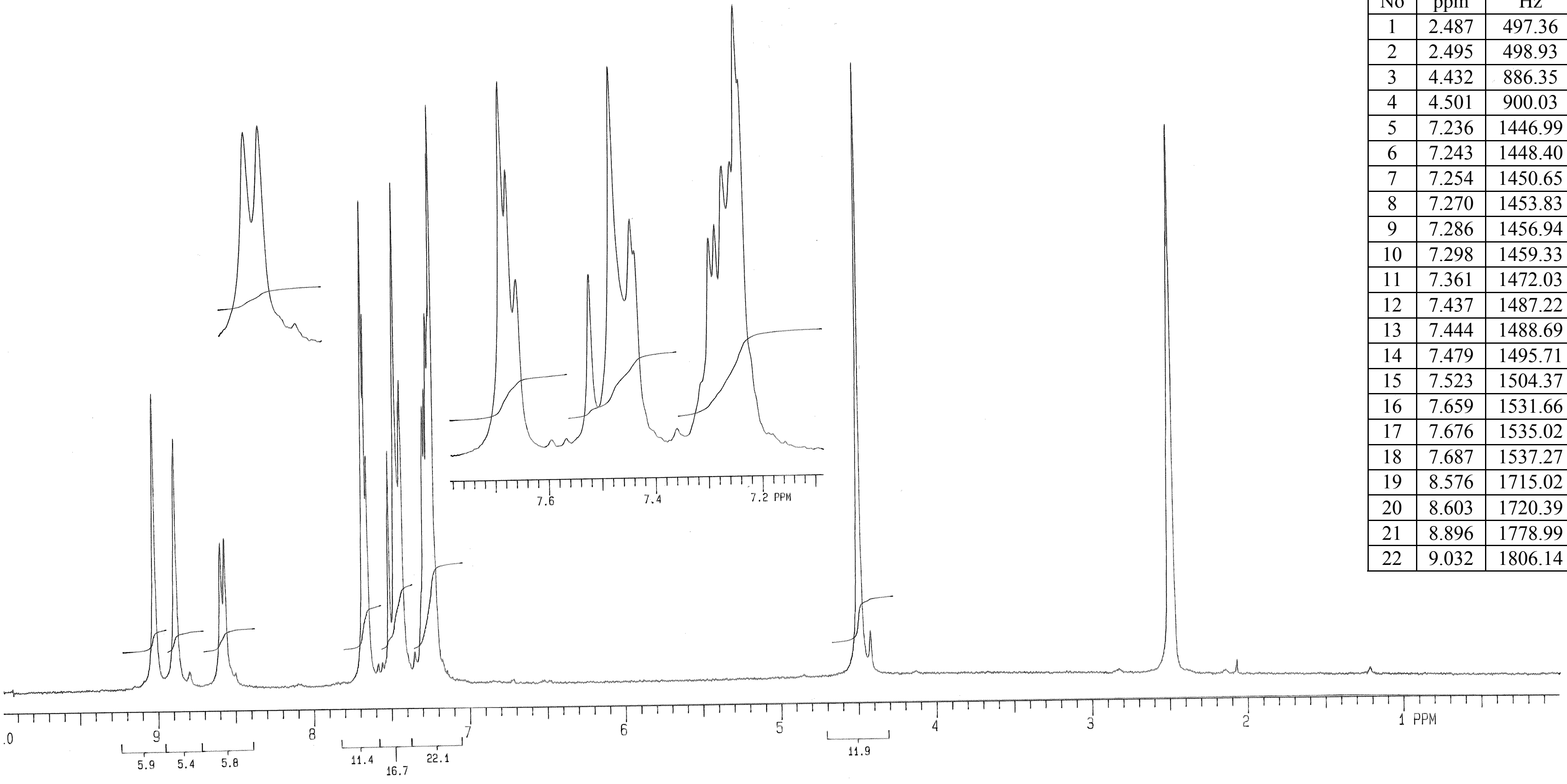

Spectrum S3. ^{1}H-NMR of compound **13** (200 MHz, DMSO-d_6).

No	ppm	Hz	intensity
1	35.204	1770.4	43.330
2	38.544	1938.4	28.761
3	38.963	1959.4	930.343
4	39.208	1971.7	9.379
5	39.382	1980.5	183.847
6	39.650	1994.0	16.620
7	39.796	2001.3	218.328
8	40.065	2014.8	19.448
9	40.215	2022.4	185.845
10	40.473	2035.4	13.532
11	40.633	2043.4	95.727
12	41.048	2064.3	29.536
13	119.316	6000.3	52.625
14	120.268	6048.2	52.335
15	121.152	6092.6	40.259
16	124.829	6277.6	18.814
17	127.905	6432.3	60.099
18	128.867	6480.7	131.040
19	129.486	6511.8	128.652
20	130.911	6583.5	54.287
21	131.342	6605.1	32.519
22	132.129	6644.7	11.712
23	135.200	6799.1	49.661
24	138.621	6971.2	33.141
25	149.707	7528.7	20.588
26	150.109	7548.9	18.732
27	150.322	7559.6	14.226
28	151.363	7612.0	10.461
29	151.409	7614.3	12.373
30	151.490	7618.4	14.816
31	151.598	7623.7	7.267

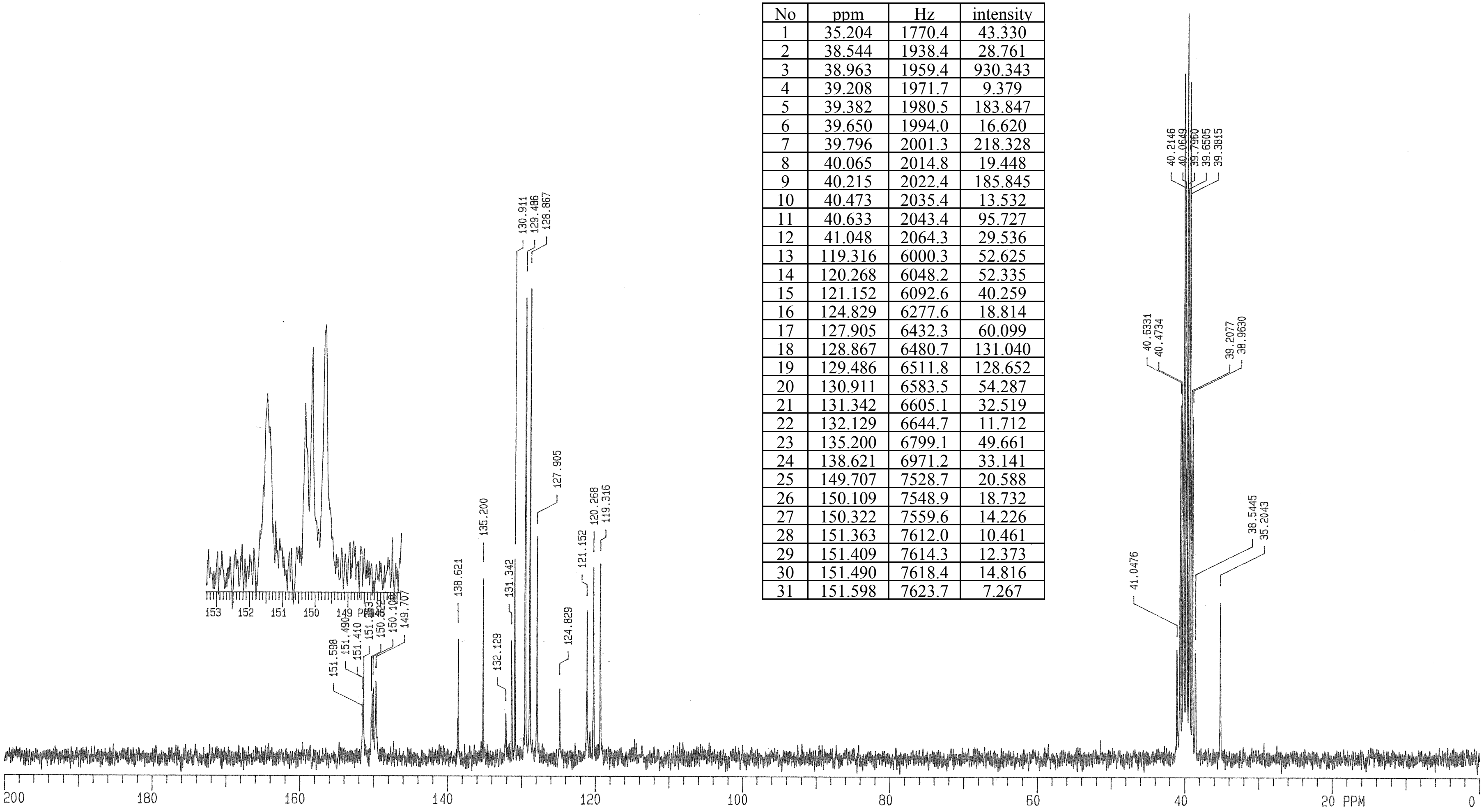

Spectrum S4. ^{13}C-NMR of compound **13** (50 MHz, DMSO-d_6).

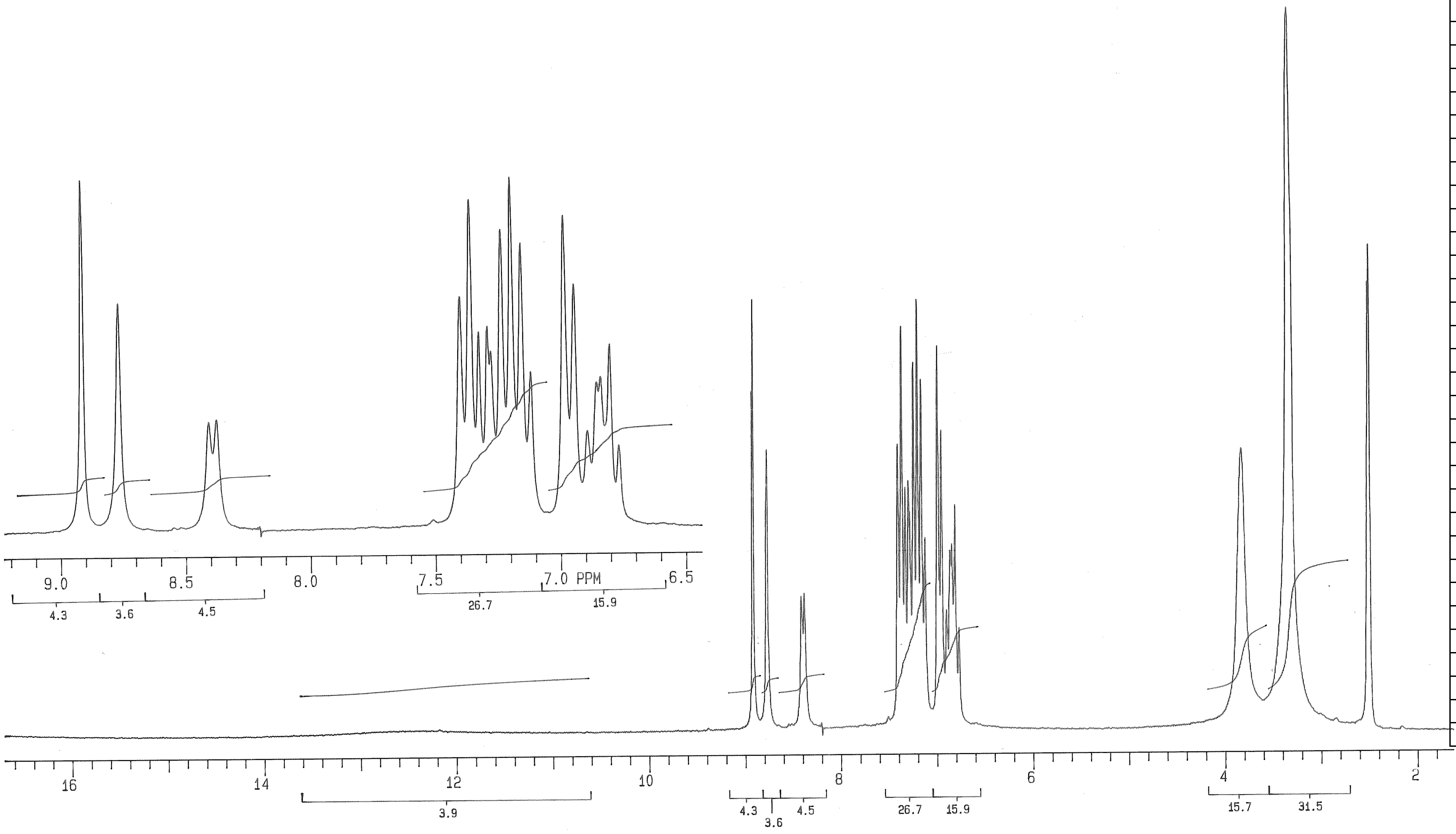

No	ppm	Hz	intensity
1	2.502	500.29	118.807
2	3.125	624.94	6.610
3	3.331	666.12	176.996
4	3.541	708.16	8.778
5	3.609	721.74	7.243
6	3.623	724.60	7.335
7	3.631	726.10	7.527
8	3.641	728.09	7.823
9	3.646	729.12	7.971
10	3.653	730.58	8.122
11	3.825	764.91	69.176
12	6.767	1353.21	25.648
13	6.803	1360.36	55.891
14	6.839	1367.62	46.322
15	6.856	1370.94	44.698
16	6.892	1378.32	30.062
17	6.945	1388.75	74.010
18	6.985	1396.89	94.637
19	7.054	1410.58	5.813
20	7.061	1412.04	5.783
21	7.118	1423.45	47.880
22	7.157	1431.29	86.503
23	7.197	1439.23	106.185
24	7.236	1447.07	90.608
25	7.278	1455.32	54.119
26	7.291	1458.11	61.735
27	7.326	1464.96	60.038
28	7.361	1471.97	99.540
29	7.401	1480.02	70.665
30	8.377	1675.11	34.424
31	8.408	1681.34	33.686
32	8.769	1753.56	69.603
33	8.915	1782.87	106.376

Spectrum S5. ^{1}H-NMR of compound **15** (200 MHz, DMSO-d_6).

No	ppm	Hz	Intensity
1	38.540	1938.2	29.736
2	38.959	1959.2	92.123
3	39.377	1980.3	184.801
4	39.794	2001.2	218.468
5	40.065	2014.8	20.076
6	40.211	2022.2	186.038
7	40.477	2035.6	14.441
8	40.629	2043.2	94.087
9	40.904	2057.0	7.044
10	41.046	2064.2	32.521
11	48.140	2421.0	51.165
12	51.060	2567.8	40.435
13	114.350	5750.6	12.506
14	115.676	5817.3	84.545
15	118.309	5949.7	60.120
16	119.342	6001.6	32.524
17	121.611	6115.7	16.297
18	128.718	6473.1	72.865
19	129.154	6495.1	28.259
20	129.288	6501.8	85.319
21	140.590	7070.2	8.763
22	150.817	7584.5	28.834
23	156.687	7879.7	22.691

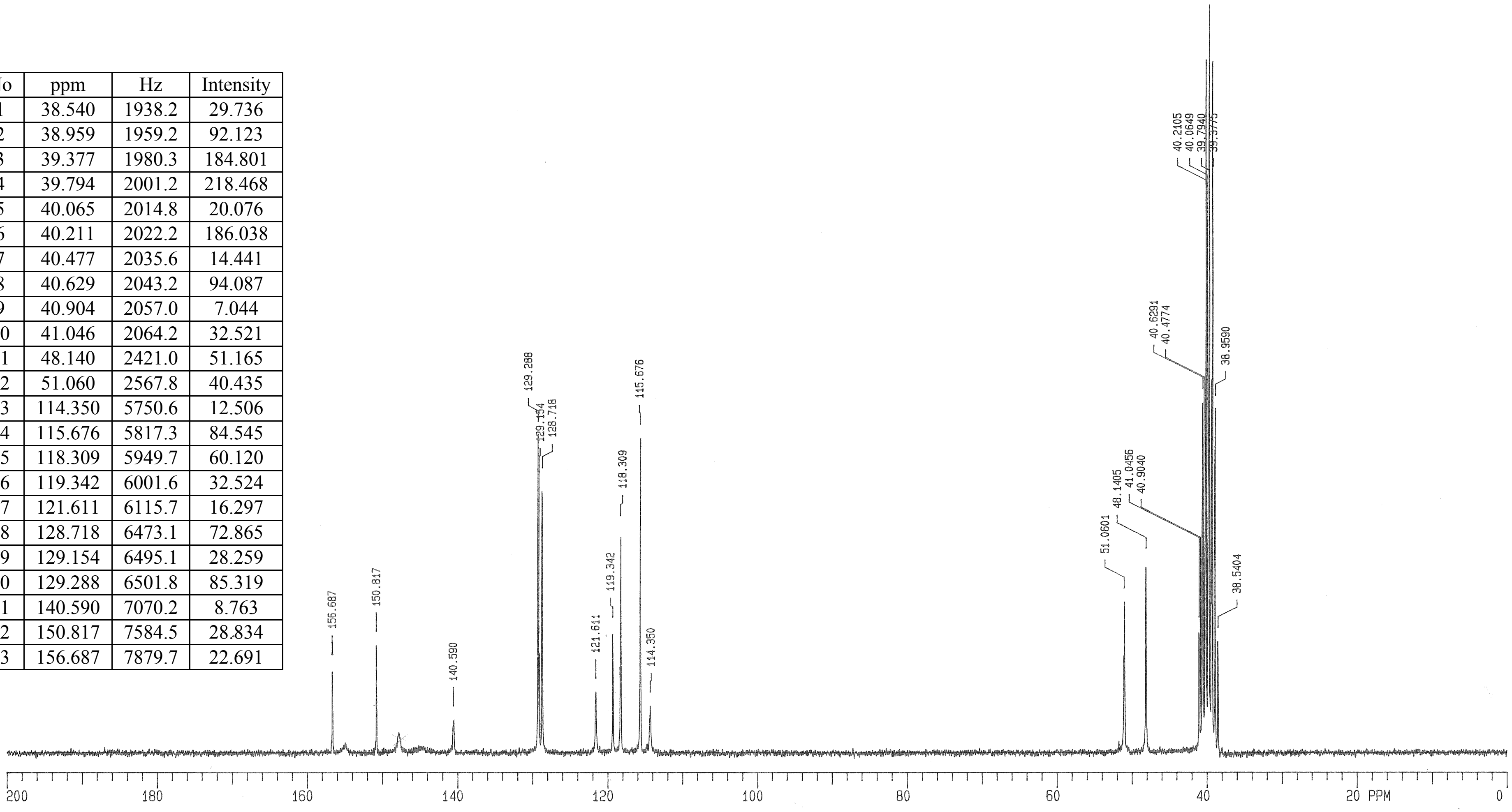

Spectrum S6. ^{13}C-NMR of compound **15** (50 MHz, DMSO-d_6).

No	ppm	Hz	intensity
1	2.495	498.93	115.355
2	3.293	658.61	123.672
3	4.026	805.10	110.571
4	6.757	1351.23	25.538
5	6.792	1358.31	57.992
6	6.829	1365.63	36.016
7	6.914	1382.72	78.910
8	6.957	1391.21	106.633
9	7.184	1436.68	70.463
10	7.222	1444.25	97.914
11	7.258	1451.33	91.998
12	7.292	1458.17	66.867
13	7.316	1463.05	94.192
14	7.326	1465.06	186.406
15	7.844	1568.52	82.555
16	7.847	1569.20	85.224
17	8.261	1652.03	53.466
18	8.297	1659.11	50.218
19	8.884	1776.54	127.083
20	9.118	1823.42	85.558

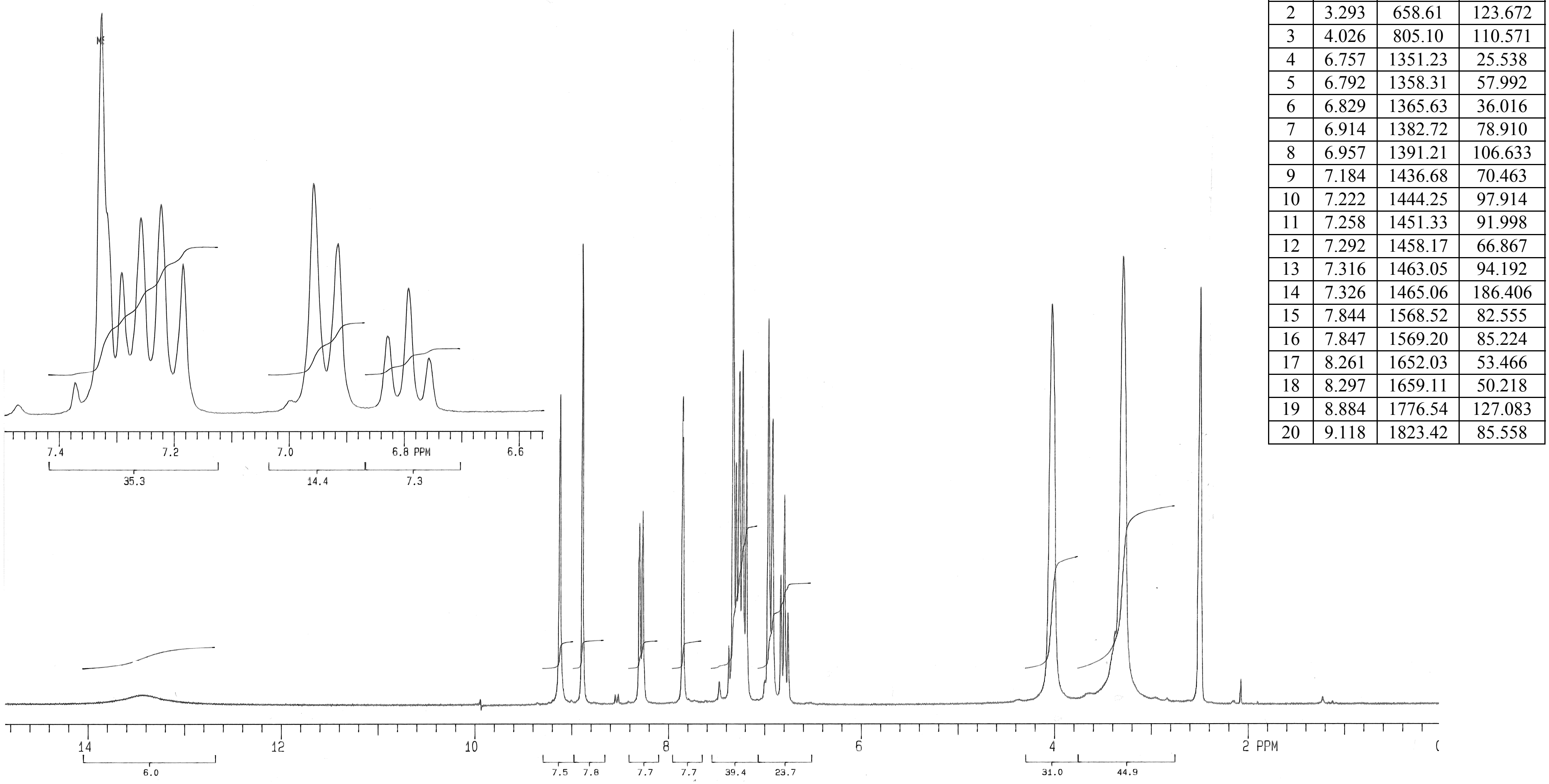

Spectrum S7. ^{1}H-NMR of compound **17** (200 MHz, DMSO-d_6).

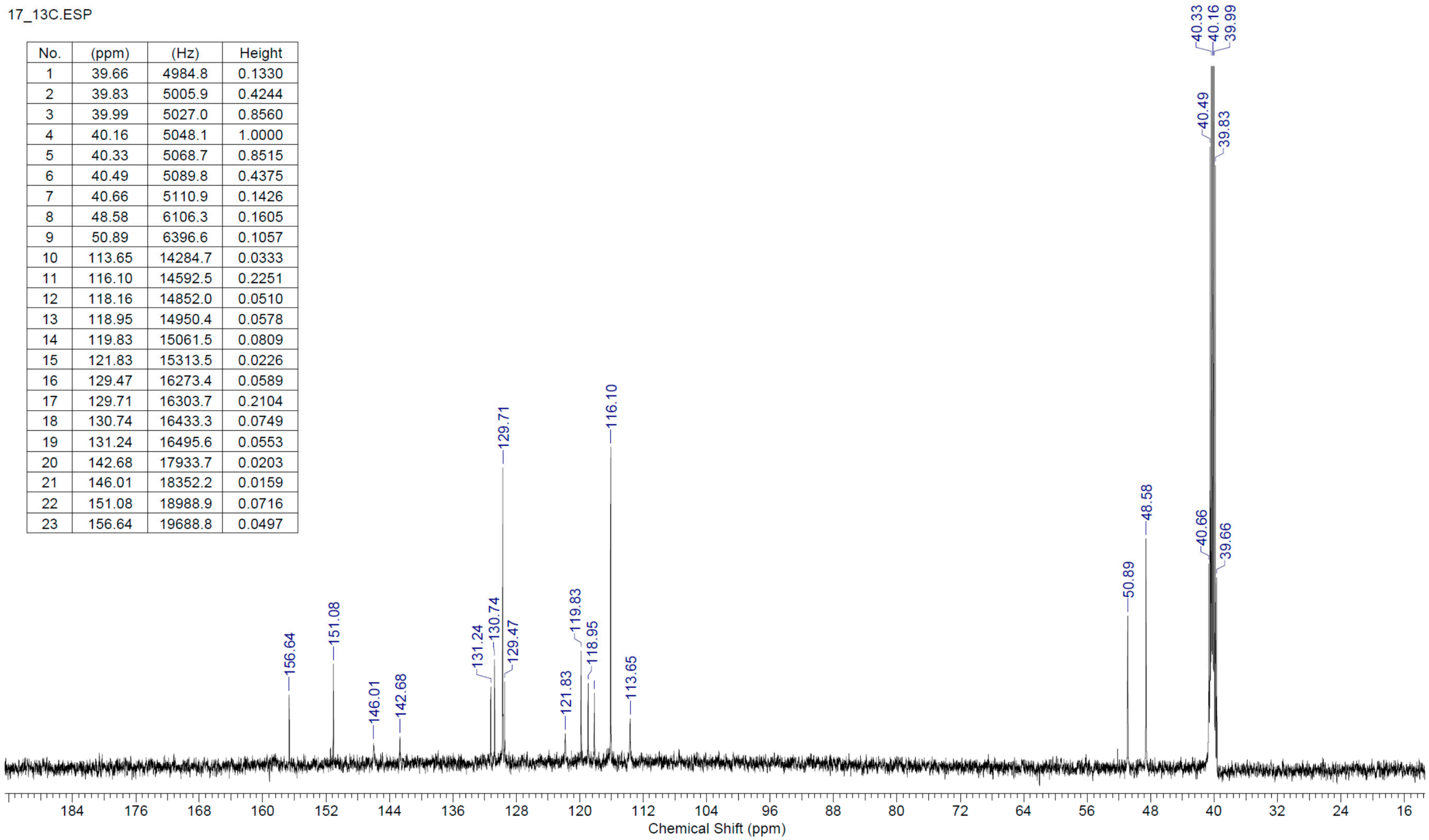

No.	(ppm)	(Hz)	Height
1	39.66	4984.8	0.1330
2	39.83	5005.9	0.4244
3	39.99	5027.0	0.8560
4	40.16	5048.1	1.0000
5	40.33	5068.7	0.8515
6	40.49	5089.8	0.4375
7	40.66	5110.9	0.1426
8	48.58	6106.3	0.1605
9	50.89	6396.6	0.1057
10	113.65	14284.7	0.0333
11	116.10	14592.5	0.2251
12	118.16	14852.0	0.0510
13	118.95	14950.4	0.0578
14	119.83	15061.5	0.0809
15	121.83	15313.5	0.0226
16	129.47	16273.4	0.0589
17	129.71	16303.7	0.2104
18	130.74	16433.3	0.0749
19	131.24	16495.6	0.0553
20	142.68	17933.7	0.0203
21	146.01	18352.2	0.0159
22	151.08	18988.9	0.0716
23	156.64	19688.8	0.0497

Spectrum S8. ^{13}C-NMR of compound **17** (125 MHz, DMSO-d_6).

No	ppm	Hz	intensity
1	2.503	500.45	95.952
2	3.379	675.67	131.218
3	3.932	786.29	67.691
4	6.916	1383.10	23.966
5	6.928	1385.47	25.266
6	6.961	1392.07	29.505
7	6.974	1394.56	30.504
8	7.146	1429.01	122.598
9	7.191	1437.93	91.952
10	7.265	1452.88	40.632
11	7.300	1459.73	43.013
12	7.388	1477.37	64.418
13	7.415	1482.72	99.469
14	7.432	1486.13	67.599
15	7.458	1491.48	68.872
16	8.318	1663.49	31.778
17	8.352	1670.18	30.364
18	8.893	1778.31	90.587
19	8.923	1784.33	63.536

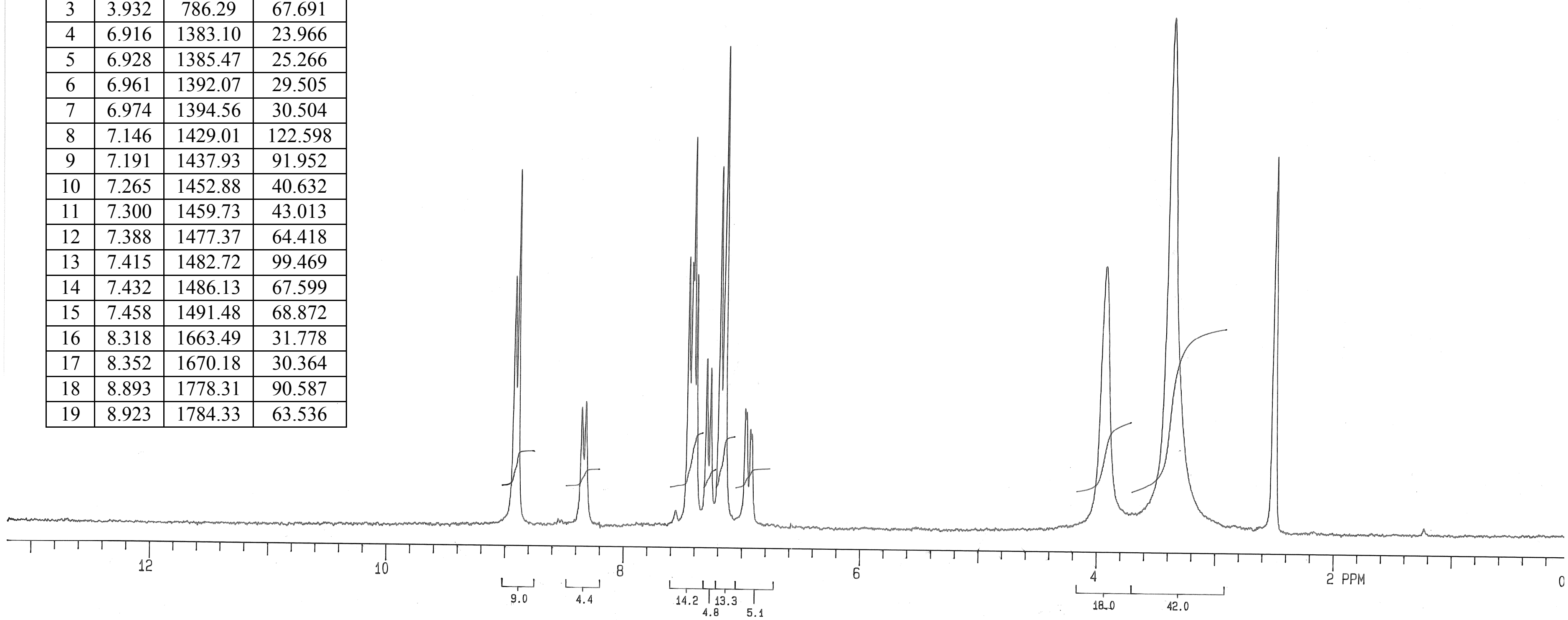

Spectrum S9. ^{1}H-NMR of compound **21** (200 MHz, DMSO-d_6).

No	ppm	Hz	intensity
1	38.544	1938.4	35.231
2	38.961	1959.3	103.200
3	39.380	1980.4	207.874
4	39.796	2001.3	248.844
5	40.065	2014.8	20.979
6	40.215	2022.4	210.071
7	40.469	2035.2	14.039
8	40.633	2043.4	104.672
9	41.052	2064.5	35.394
10	47.316	2379.5	37.034
11	50.332	2531.2	29.034
12	113.709	5718.4	10.899
13	113.749	5720.4	11.834
14	115.440	5805.4	34.258
15	116.416	5854.5	35.955
16	119.467	6007.9	42.899
17	119.993	6034.4	24.819
18	124.431	6257.6	9.162
19	128.420	6458.2	59.621
20	129.284	6501.6	24.200
21	130.778	6576.7	33.827
22	131.817	6629.0	26.566
23	140.321	7056.7	8.827
24	150.407	7563.9	29.971
25	156.495	7870.0	26.261

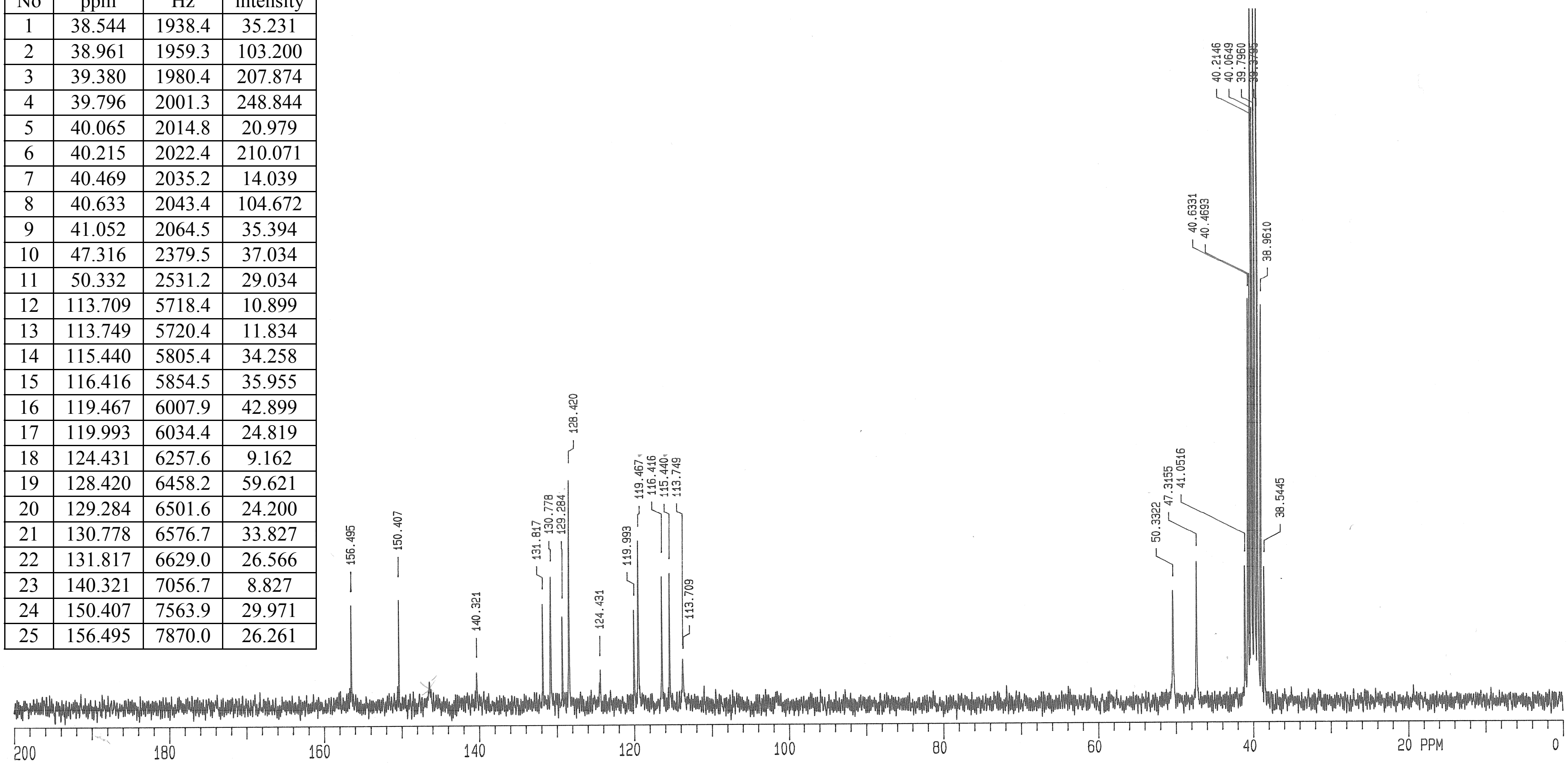

Spectrum S10. ^{13}C-NMR of compound **21** (50 MHz, DMSO-d_6).

No	ppm	Hz	Intensity
1	1.931	386.10	120.803
2	2.052	410.38	123.727
3	2.168	433.63	131.093
4	2.503	500.45	38.373
5	3.349	669.65	15.722
6	7.010	1401.87	10.526
7	7.041	1408.00	19.407
8	7.072	1414.32	14.081
9	7.243	1448.36	16.516
10	7.285	1456.77	56.916
11	7.316	1463.00	119.066
12	7.561	1512.03	30.934
13	7.587	1517.22	30.541
14	8.947	1789.26	31.839
15	8.973	1794.45	30.893
16	9.080	1815.71	40.782
17	9.270	1853.69	50.654

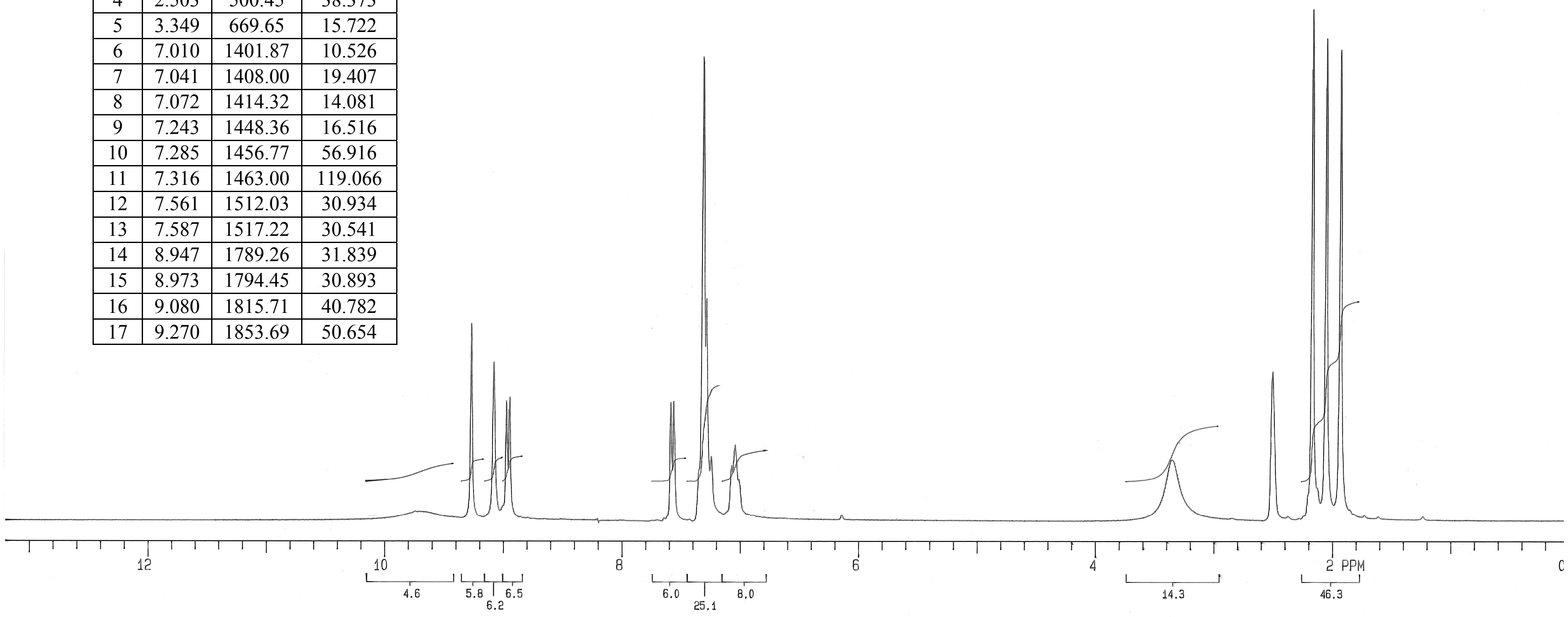

Spectrum S11. ^{1}H-NMR of compound **24** (200 MHz, DMSO-d_6).

24_13C.ESP

No.	(ppm)	(Hz)	Height
1	8.56	1075.5	0.1636
2	10.61	1333.2	0.1814
3	12.64	1588.8	0.1938
4	39.66	4985.3	0.1321
5	39.83	5005.9	0.4208
6	39.99	5027.0	0.8535
7	40.16	5048.1	1.0000
8	40.33	5069.1	0.8540
9	40.50	5090.2	0.4363
10	40.66	5110.9	0.1484
11	114.15	14347.9	0.1207
12	119.43	15011.0	0.4032
13	124.07	15594.6	0.1635
14	124.21	15612.1	0.1795
15	129.65	16296.3	0.4448
16	132.08	16601.5	0.0865
17	138.48	17405.5	0.2349
18	144.97	18221.3	0.1083
19	149.67	18812.8	0.0962
20	150.12	18869.4	0.1126
21	152.88	19216.3	0.1724
22	155.93	19599.2	0.1571

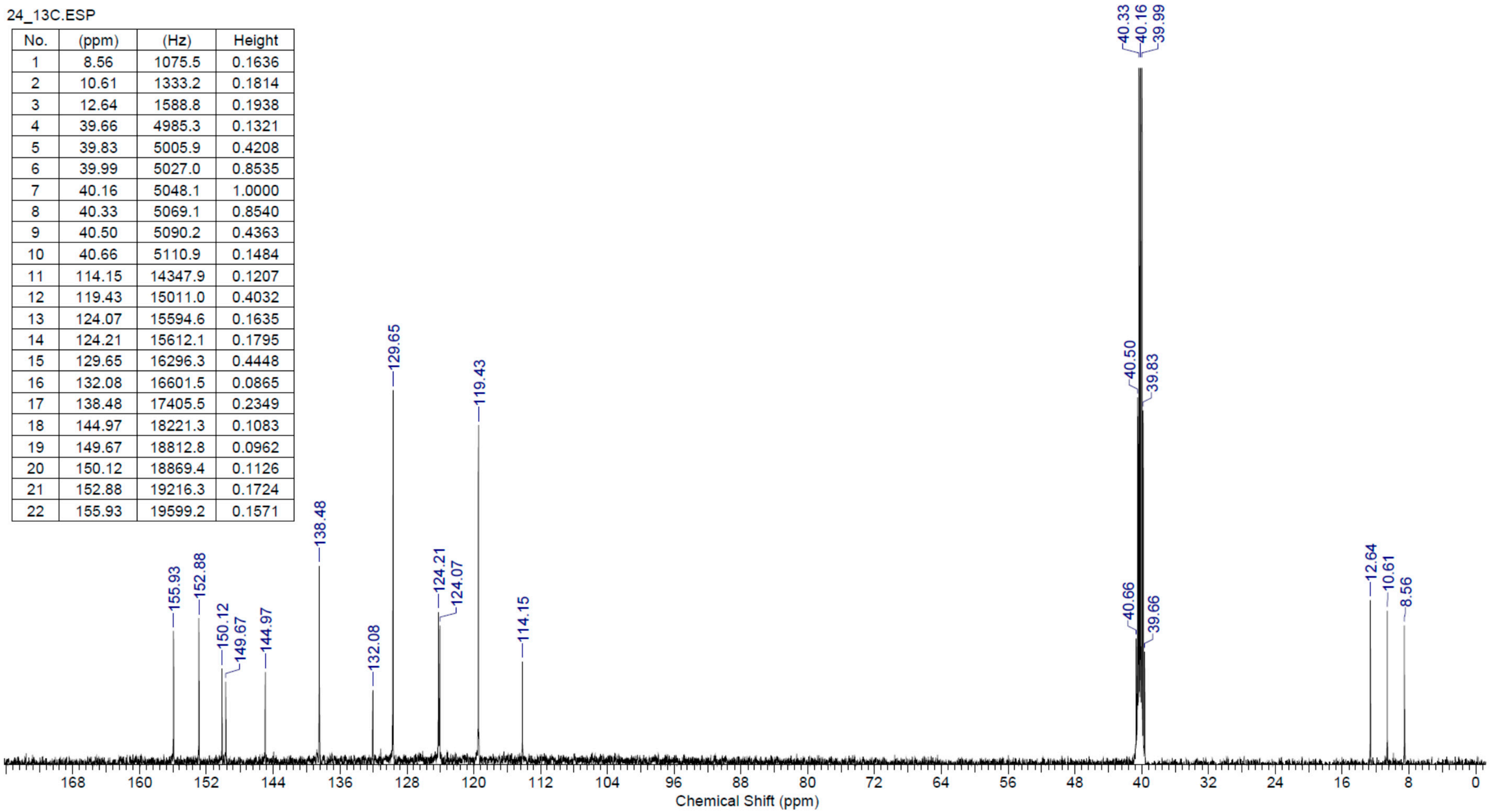

Spectrum S12. ^{13}C-NMR of compound **24** (125 MHz, DMSO-d_6).

No	ppm	Hz	intensity
1	1.927	385.37	102.111
2	2.050	409.91	103.528
3	2.155	430.99	131.541
4	2.193	438.56	8.662
5	2.481	496.11	9.210
6	2.490	497.94	21.136
7	2.499	499.77	31.590
8	2.508	501.61	26.162
9	7.348	1469.43	185.925
10	7.555	1510.90	27.767
11	7.582	1516.24	29.643
12	8.944	1788.55	36.418
13	8.970	1793.76	35.655
14	9.209	1841.67	27.282
15	9.262	1852.13	50.132

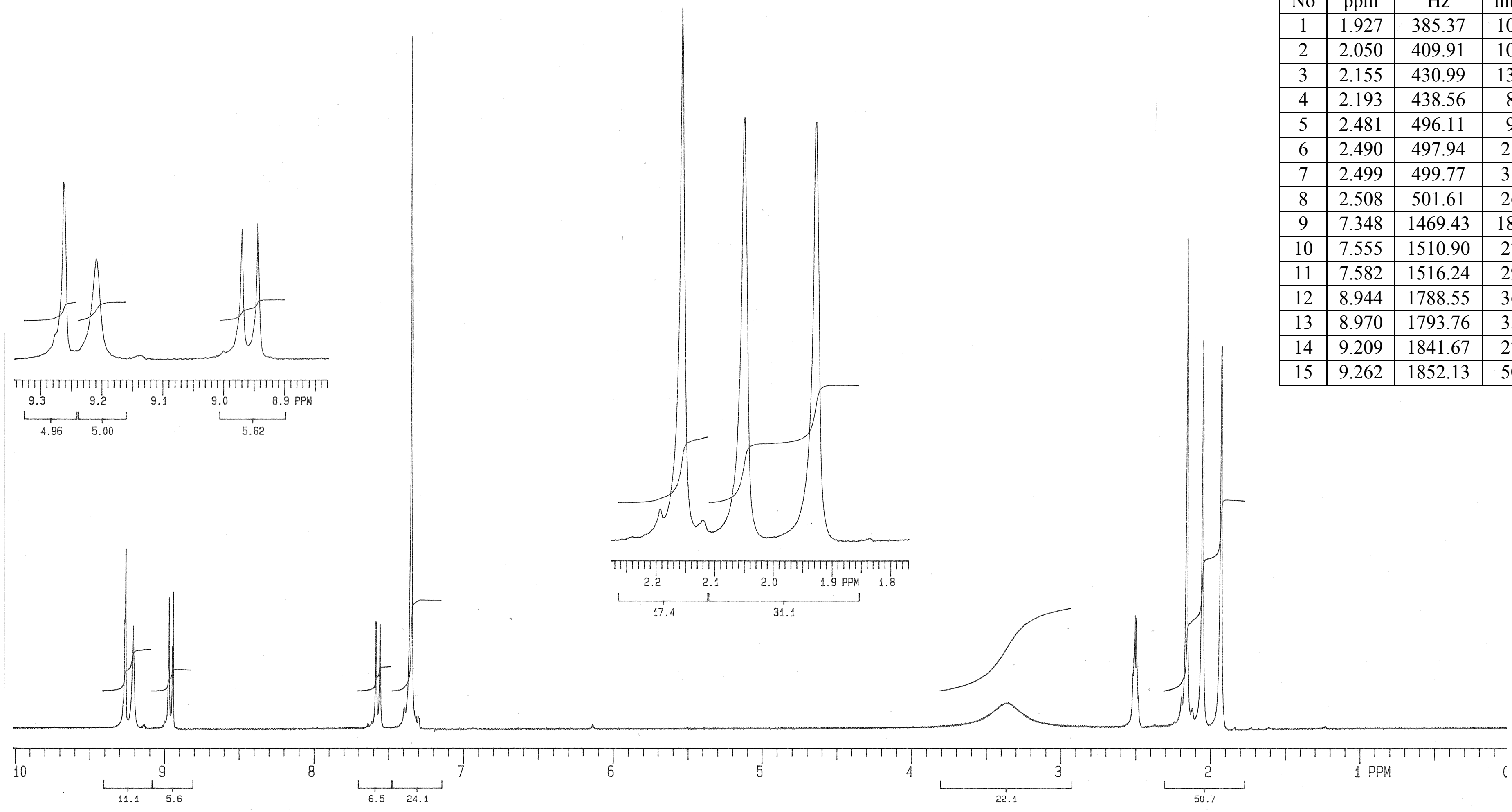

Spectrum S13. ^{1}H-NMR of compound **25** (200 MHz, DMSO-d_6).

No	ppm	Hz	intensity
1	8.131	408.9	34.325
2	10.189	512.4	41.966
3	12.209	614.0	38.650
4	38.540	1938.2	22.176
5	38.955	1959.0	68.073
6	39.373	1980.1	137.534
7	39.792	2001.1	161.506
8	40.069	2015.0	13.712
9	40.211	2022.2	139.638
10	40.625	2043.0	70.649
11	41.048	2064.3	24.787
12	113.755	5720.7	21.970
13	120.693	6069.6	82.095
14	123.829	6227.3	34.772
15	127.290	6401.3	22.402
16	129.108	6492.8	87.818
17	131.641	6620.2	18.071
18	137.070	6893.2	30.477
19	138.093	6944.6	24.193
20	144.565	7270.1	21.589
21	149.282	7507.3	27.947
22	149.725	7529.6	24.259
23	152.404	7664.3	35.611
24	155.567	7823.3	35.997

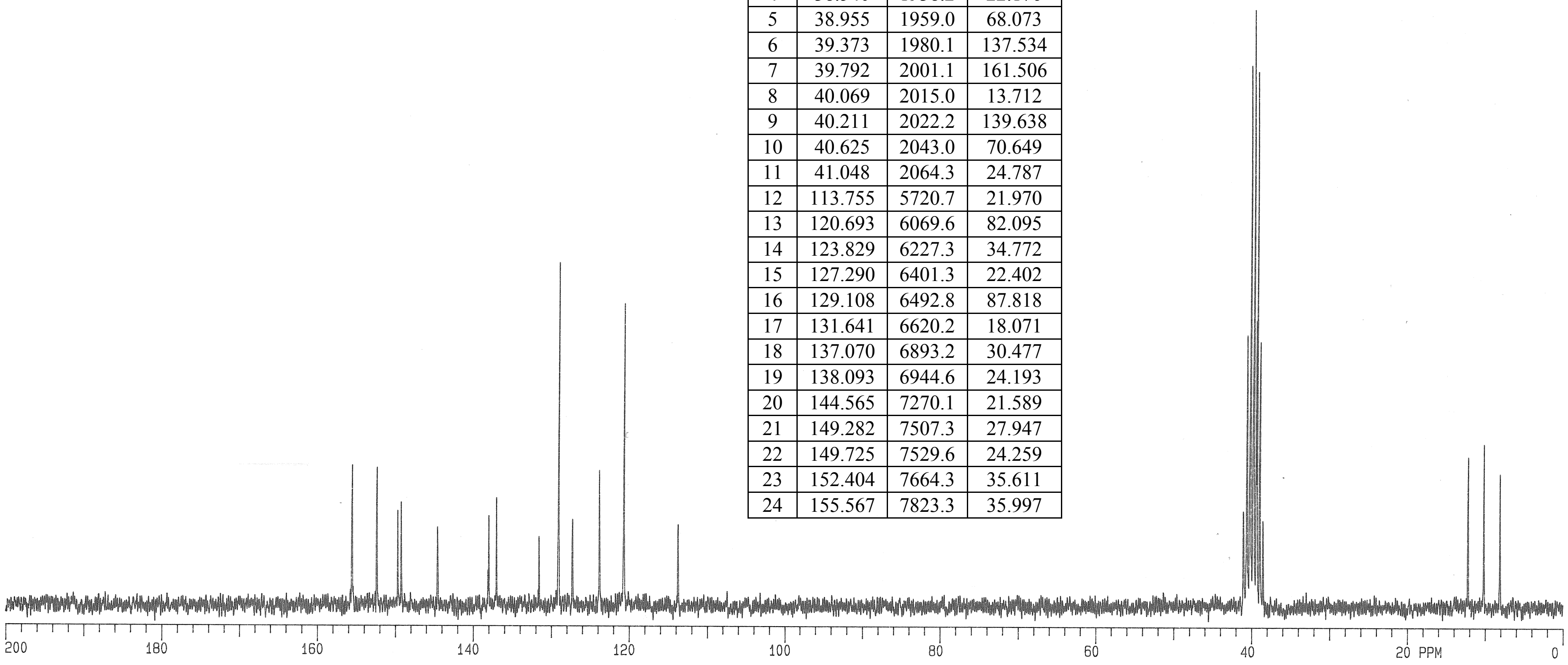

Spectrum S14. ^{13}C-NMR of compound **25** (50 MHz, DMSO-d_6).

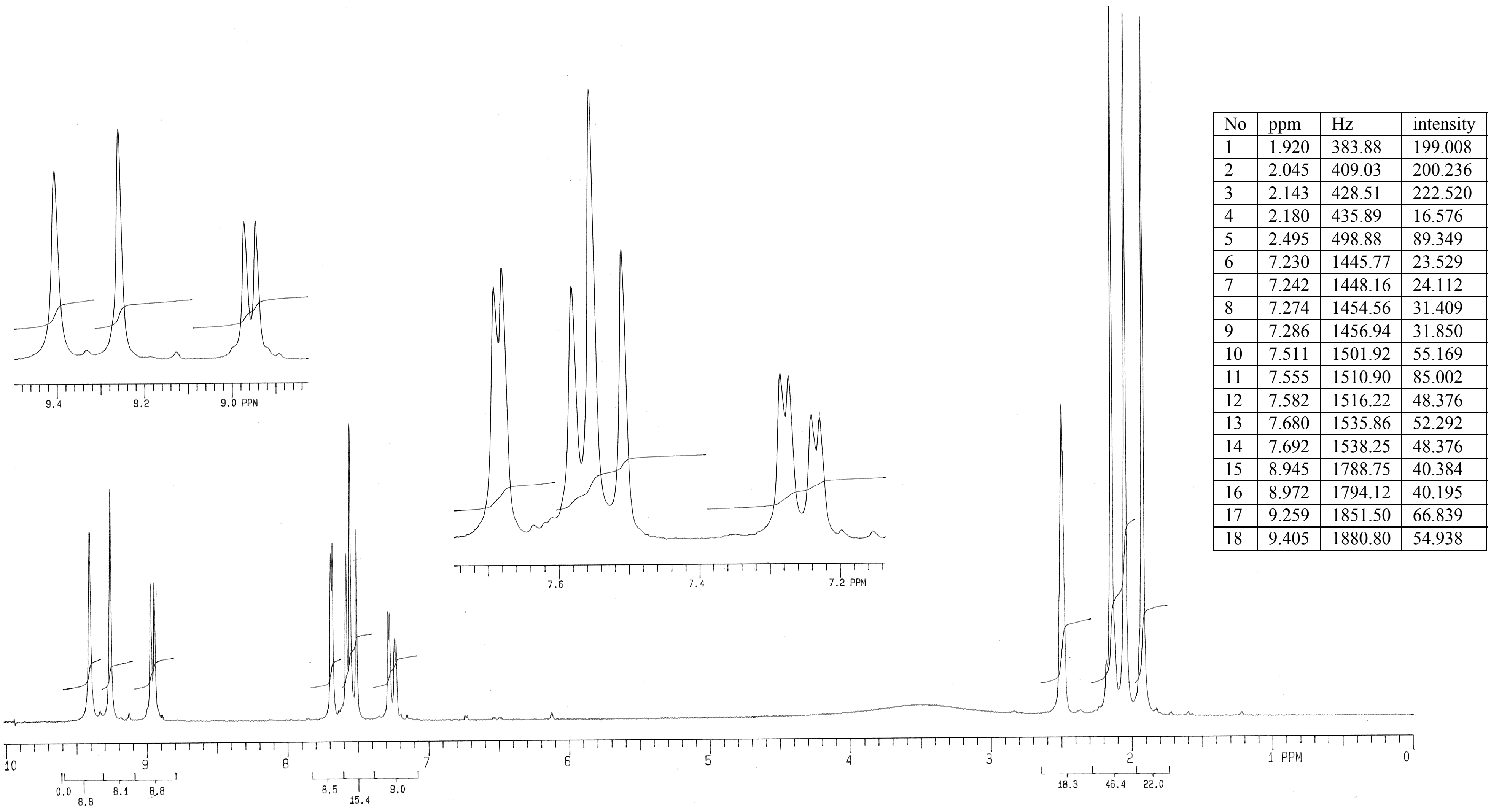

No	ppm	Hz	intensity
1	1.920	383.88	199.008
2	2.045	409.03	200.236
3	2.143	428.51	222.520
4	2.180	435.89	16.576
5	2.495	498.88	89.349
6	7.230	1445.77	23.529
7	7.242	1448.16	24.112
8	7.274	1454.56	31.409
9	7.286	1456.94	31.850
10	7.511	1501.92	55.169
11	7.555	1510.90	85.002
12	7.582	1516.22	48.376
13	7.680	1535.86	52.292
14	7.692	1538.25	48.376
15	8.945	1788.75	40.384
16	8.972	1794.12	40.195
17	9.259	1851.50	66.839
18	9.405	1880.80	54.938

Spectrum S15. ^{1}H-NMR of compound **26** (200 MHz, DMSO-d_6).

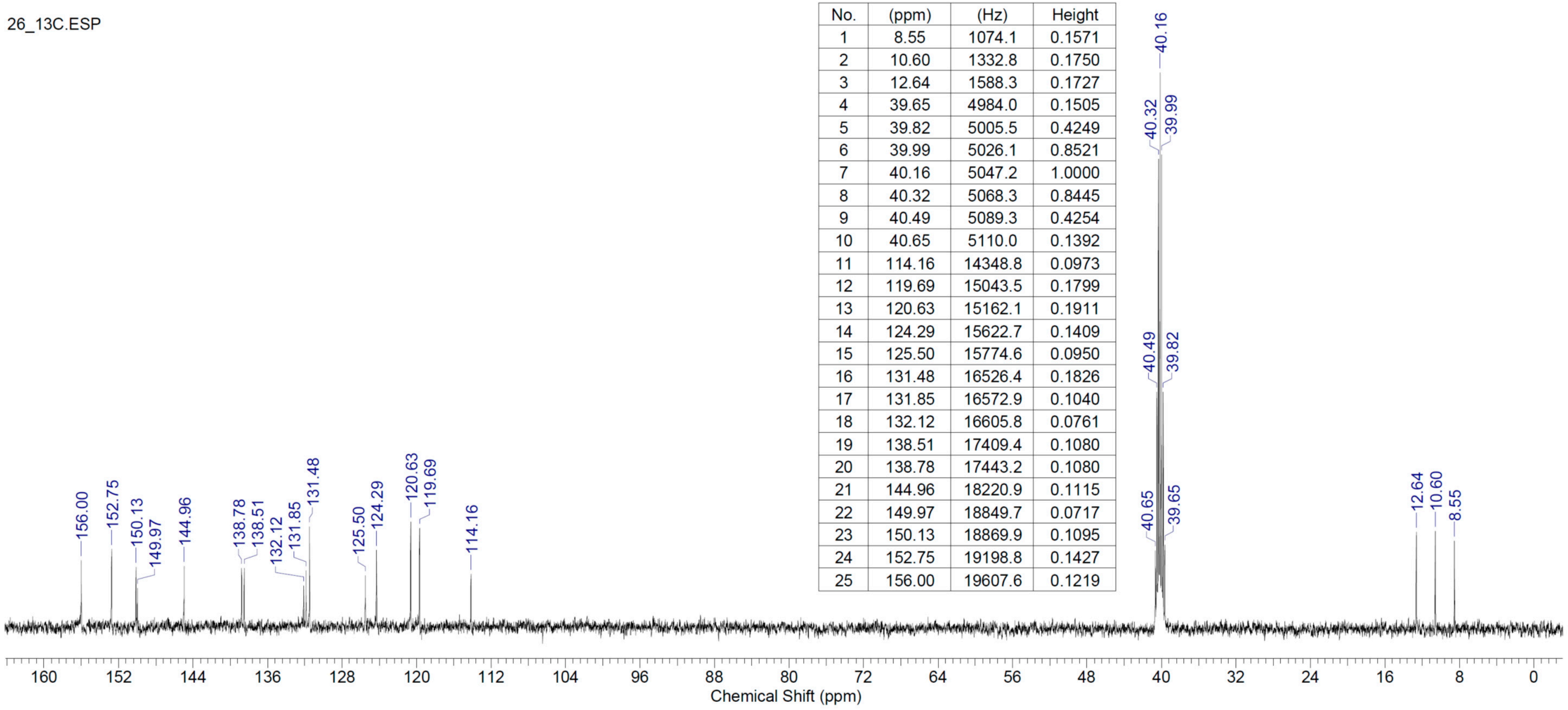

No.	(ppm)	(Hz)	Height
1	8.55	1074.1	0.1571
2	10.60	1332.8	0.1750
3	12.64	1588.3	0.1727
4	39.65	4984.0	0.1505
5	39.82	5005.5	0.4249
6	39.99	5026.1	0.8521
7	40.16	5047.2	1.0000
8	40.32	5068.3	0.8445
9	40.49	5089.3	0.4254
10	40.65	5110.0	0.1392
11	114.16	14348.8	0.0973
12	119.69	15043.5	0.1799
13	120.63	15162.1	0.1911
14	124.29	15622.7	0.1409
15	125.50	15774.6	0.0950
16	131.48	16526.4	0.1826
17	131.85	16572.9	0.1040
18	132.12	16605.8	0.0761
19	138.51	17409.4	0.1080
20	138.78	17443.2	0.1080
21	144.96	18220.9	0.1115
22	149.97	18849.7	0.0717
23	150.13	18869.9	0.1095
24	152.75	19198.8	0.1427
25	156.00	19607.6	0.1219

Spectrum S16. ^{13}C-NMR of compound **26** (125 MHz, DMSO-d_6).

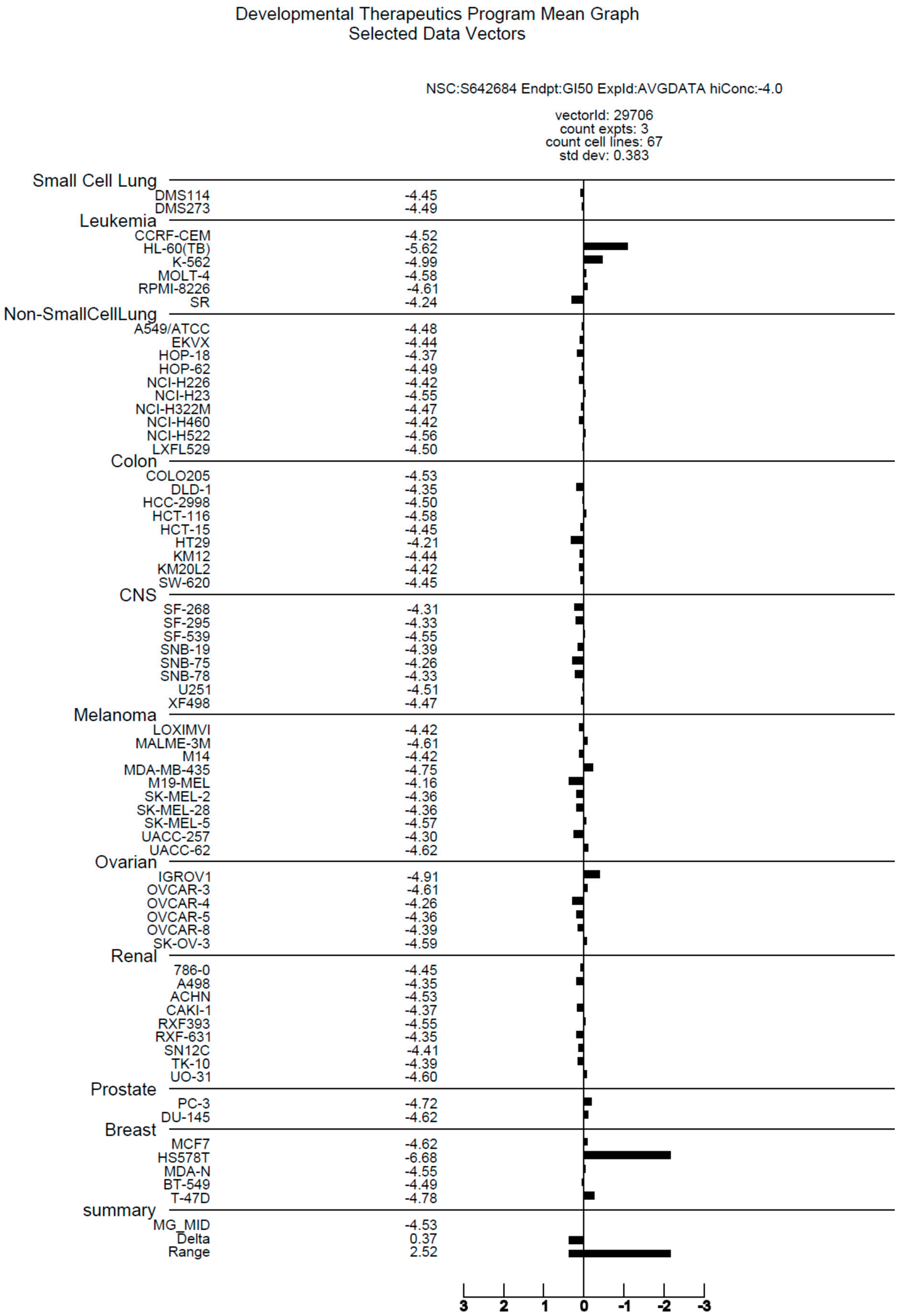

Graph S1. NCI Cancer Screen Current Data—DTP 60 cell/5 dose for Sulofenur (NSC-642684)—GI$_{50}$ [log10(M)].

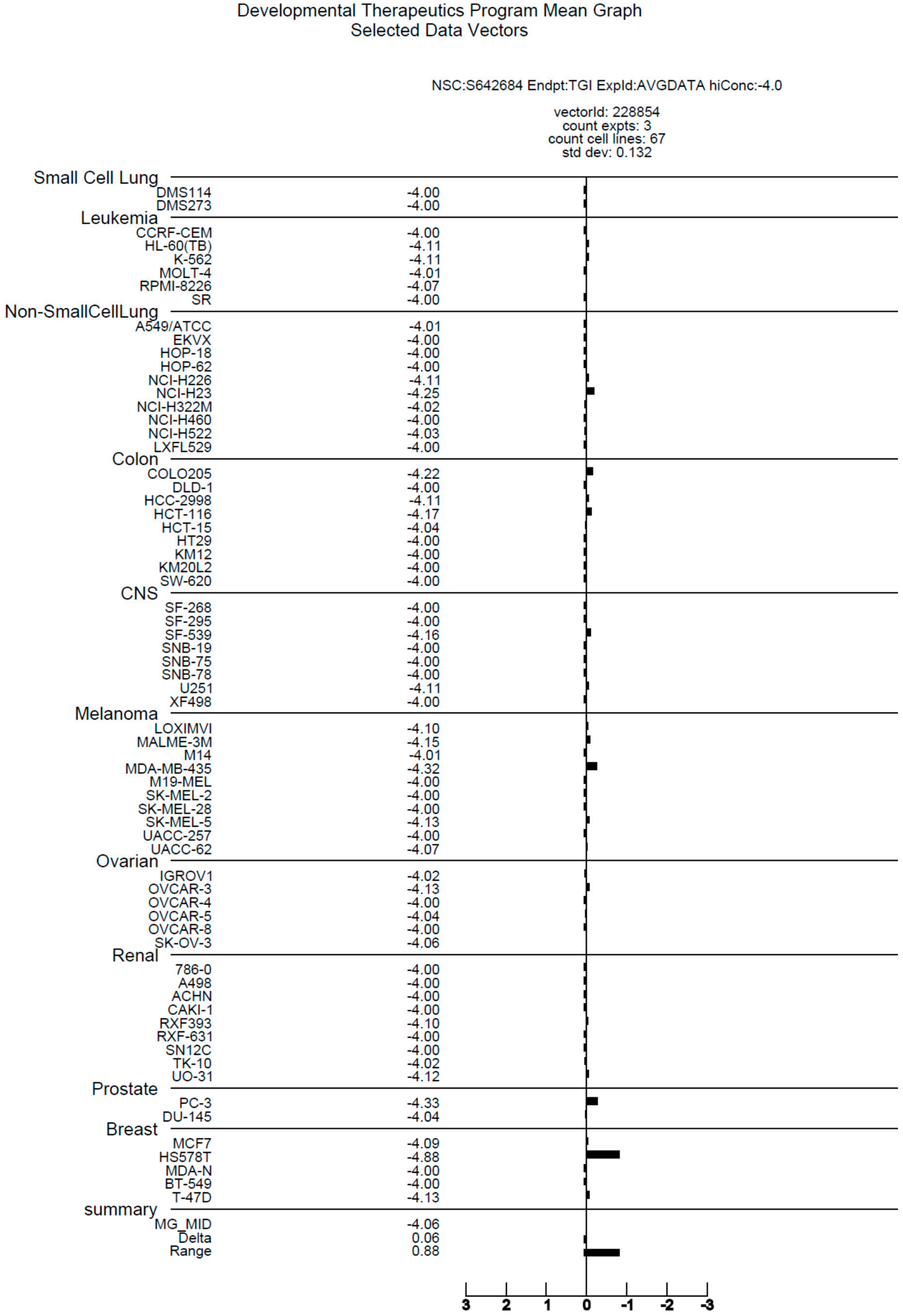

Graph S2. NCI Cancer Screen Current Data—DTP 60 cell/5 dose for Sulofenur (NSC-642684)—TGI [log10(M)].

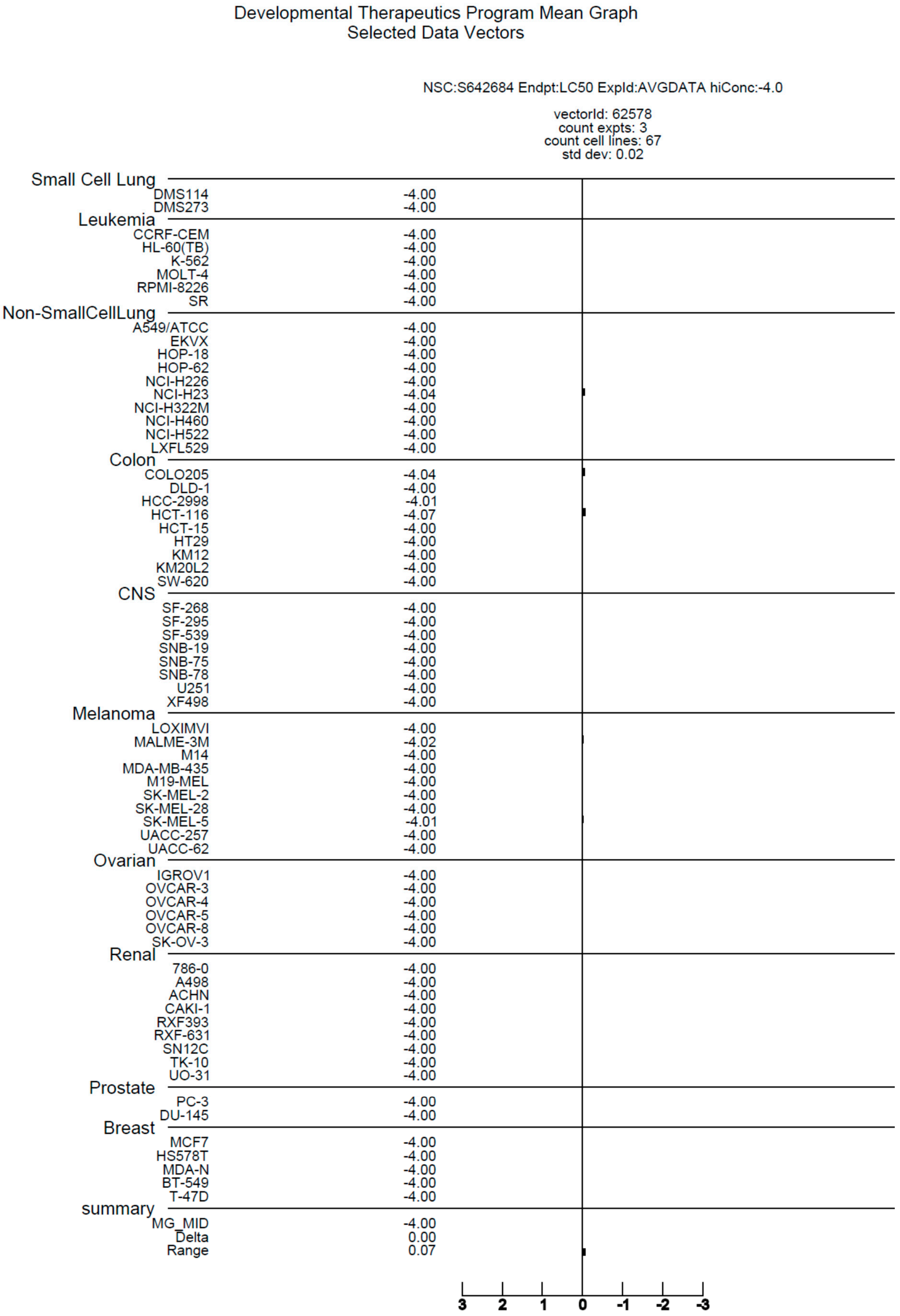

Graph S3. NCI Cancer Screen Current Data—DTP 60 cell/5 dose for Sulofenur (NSC-642684)—LC_{50} [log10(M)].

References

1. Alley, M.C.; Scudiero, D.A.; Monks, P.A.; Hursey, M.L.; Czerwinski, M.J.; Fine, D.L.; Abbott, B.J.; Mayo, J.G.; Shoemaker, R.H.; Boyd, M.R. Feasibility of Drug Screening with Panels of Human Tumor Cell Lines Using a Microculture Tetrazolium Assay. *Cancer Res*. **1988**, *48*, 589–601.
2. Grever, M.R.; Schepartz, S.A.; Chabner, B.A. The National Cancer Institute: Cancer Drug Discovery and Development Program. *Semin. Oncol.* **1992**, *19*, 622–638.
3. Boyd, M.R.; Paull, K.D. Some Practical Considerations and Applications of the National Cancer Institute In Vitro Anticancer Drug Discovery Screen. *Drug Dev. Res.* **1995**, *34*, 91–109.
4. Shoemaker, R.H. The NCI60 Human Tumour Cell line Anticancer Drug Screen. *Nat. Rev*. **2006**, *6*, 813–823.
5. NCI-60 DTP Human Tumor Cell Line Screen. Available online: http://dtp.nci.nih.gov/branches/btb/ivclsp.html (accessed on 7 May 2015).
